# Human Sirtuin Regulators: The “Success” Stories

**DOI:** 10.3389/fphys.2021.752117

**Published:** 2021-10-21

**Authors:** Alyson M. Curry, Dawanna S. White, Dickson Donu, Yana Cen

**Affiliations:** ^1^Department of Medicinal Chemistry, Virginia Commonwealth University, Richmond, VA, United States; ^2^Institute for Structural Biology, Drug Discovery and Development, Virginia Commonwealth University, Richmond, VA, United States

**Keywords:** sirtuin, inhibitor, activator, clinical trial, drug development

## Abstract

The human sirtuins are a group of NAD^+^-dependent protein deacylases. They “erase” acyl modifications from lysine residues in various cellular targets including histones, transcription factors, and metabolic enzymes. Through these far-reaching activities, sirtuins regulate a diverse array of biological processes ranging from gene transcription to energy metabolism. Human sirtuins have been intensely pursued by both academia and industry as therapeutic targets for a broad spectrum of diseases such as cancer, neurodegenerative diseases, and metabolic disorders. The last two decades have witnessed a flood of small molecule sirtuin regulators. However, there remain relatively few compounds targeting human sirtuins in clinical development. This reflects the inherent issues concerning the development of isoform-selective and potent molecules with good drug-like properties. In this article, small molecule sirtuin regulators that have advanced into clinical trials will be discussed in details as “successful” examples for future drug development. Special attention is given to the discovery of these compounds, the mechanism of action, pharmacokinetics analysis, formulation, as well as the clinical outcomes observed in the trials.

## Introduction

Initially, sirtuins were classified as epigenetic “eraser” enzymes dedicated for the removal of acetyl groups from histone N*-*terminal lysine residues (Blander and Guarente, 2004; Trapp and Jung, 2006). The deacetylation of histones causes chromatin condensation, which is closely associated with transcription silencing (Shahbazian and Grunstein, 2007). Unlike the Zn^2+^-dependent histone deacetylases (HDACs), sirtuins carry out the chemical modifications in an NAD^+^-dependent fashion (Imai et al., 2000; Landry et al., 2000). Growing evidence suggests that sirtuins not only act on histone proteins, but also have other cellular targets such as transcription factors and metabolic enzymes (Sauve et al., 2006; Kosciuk et al., 2019). Furthermore, diverse catalytic activities have been uncovered for sirtuins, including, but not limited to, depropionylation, debutyrylation, desuccinylation, and de-fatty acylation (Du et al., 2011; Feldman et al., 2013; Jiang et al., 2013; Anderson et al., 2017). These pleiotropic enzymatic activities give sirtuins their far-reaching functions in maintaining genome integrity, regulating metabolism homeostasis, and promoting organismal longevity (Guarente and Picard, 2005; Cen et al., 2011; Watroba et al., 2017).

The human sirtuins, SIRT1-SIRT7, have been intensively investigated for their enzymatic activities and biological functions. There are numerous wonderful review articles highlighting the significance of these enzymes in regulating normal physiology and pathophysiology (Chang and Guarente, 2014; Herskovits and Guarente, 2014; Kumar and Lombard, 2018; Chang et al., 2020; Jaiswal et al., 2021). For example, overexpression of SIRT1 has been observed to increase carcinogenesis in prostate and thyroid tumors in mice with a deficiency of tumor suppressor PTEN (phosphatase and tensin homolog) (Herranz et al., 2013). SIRT1 also plays an important role in the development of drug resistance in chronic myeloid leukemia (CML) cells (Wang et al., 2013). It has been shown to activate error-prone DNA repair which can lead to increased incidence of genetic mutations (Wang et al., 2013). Thus, a SIRT1 inhibitor would be beneficial in combination with established chemotherapeutics to reduce drug resistance. Sirtuin activators can also play an important role in disease modulation. SIRT1 is considered to have a neuroprotective role in the brain, as it helps to regulate apoptosis and BDNF (brain-derived neurotrophic factor) expression (Luo et al., 2001; Zocchi and Sassone-Corsi, 2012). Activation of SIRT1 may be beneficial for the treatment of Alzheimer’s disease (AD), in which SIRT1 levels are typically reduced (Lutz et al., 2014). AD is characterized by the presence of amyloid plaques containing amyloid beta (Aβ) and neurofibrillary tangles (NFTs) containing hyperphosphorylated tau (Ittner and Götz, 2011). SIRT1 overexpression was shown to increase α-secretase activity, thus reducing the formation of Aβ (Endres and Fahrenholz, 2012). SIRT1 also prevents AD pathology through the deacetylation of tau (Min et al., 2010). Tau acetylation inhibits the degradation of tau and is detected in early stages of diseases with abnormal tau accumulation (Min et al., 2010). Therefore, SIRT1 serves as a possible therapeutic target for the treatment of AD.

Naturally, the development of small molecule regulators targeting human sirtuins has become a hot topic of research. Despite all the efforts over the last few decades, the success stories were scarce. Many small molecule sirtuin inhibitors and activators can only be called “chemical probes” at the present time due to the lack of isoform selectivity, moderate potency, limited bioavailability, and poor pharmacokinetic (PK) and pharmacodynamic (PD) profiles. There is a clear gap between the pre-clinical probe discovery and clinical drug candidate development. There is also a gap in the amount of research effort put into studying the various sirtuin isoforms. SIRT1 is by far the most studied isoform, with over 11,000 articles indexed in PubMed. In comparison, the other two most studied isoforms, SIRT2 and SIRT3, together have only around 3,700 articles. This disparity in research translates to fewer small molecule modulators targeting the other isoforms. Thus, the modulators that have entered clinical trials are primarily focused on SIRT1.

In this review, we will “tell the tales” of several human sirtuin regulators that have advanced into clinical investigation for the treatment of various diseases. The focus of the discussions will be the discovery of these compounds, their mechanism of action (MOA), and the rationale and outcome of the clinical trials. Although we brand these compounds as the “success” stories, they are not without controversy or limitation. On the flip side, the lessons we learn from these examples may help guide the design and development of the next generation of sirtuin regulators as therapeutic candidates.

For the benefit of the general audience, we would like to briefly discuss the basic theories behind the development of small molecule drug candidates toward clinical trials. The candidate compounds are normally small molecules, either natural products or synthetic compounds, with desired biological activity toward target proteins or enzymes in the *in vitro* setting. These candidates are the results of rounds of optimization for improved potency, selectivity, and solubility. For example, Lipinski’s rule of five (Ro5) has been the golden standard to prioritize the drug-like properties of orally active compounds (Lipinski et al., 2001). The “druggability” of the candidates will then be analyzed through ADME (absorption, distribution, metabolism and excretion) studies. These studies will assess the bioavailability, distribution, stability, and elimination of the candidate compounds. In the following sections, C_max_ of certain sirtuin modulators will be discussed. This critical parameter in ADME analysis describes the maximum concentration of a candidate compound in targeted tissue/organ after administration. The results from ADME studies will guide the further optimization of the candidate molecules. The best dosage, administration route, and formulation need to be evaluated as well. The formulations of resveratrol (RSV) will be discussed in detail in the next section. The active ingredient, RSV, has been combined with variety of substances to improve its bioavailability.

## Resveratrol and Related SRT Compounds

### Resveratrol

**FIGURE 1:**
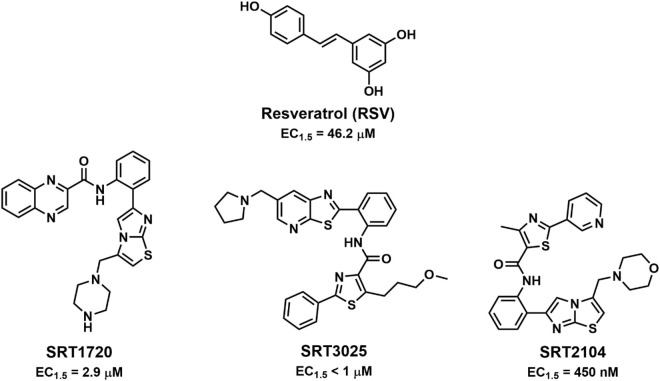
Structures of resveratrol and the SRT family of SIRT1 activators.

In the 1980s, epidemiologists observed that developed countries with increased wine consumption had decreased deaths due to ischemic heart disease (IHD) (St Leger et al., 1979). This later came to be known as the “French Paradox” because of the lowered IHD mortality rates in France despite having no difference in saturated fat intake or blood cholesterol levels (Burr, 1995). In Bertelli et al. (1995) implicated resveratrol (RSV, Figure 1) as the mediator of the cardioprotective effects of wine, thus spurring interest in the molecule as a potential therapeutic. The association between RSV and SIRT1 was discovered in 2003 by high-throughput screening (HTS) utilizing a Fluor de Lys deacetylation assay on a library of plant-derived polyphenols (Howitz et al., 2003). Howitz et al. (2003) found that RSV activated SIRT1 deacetylase activity by decreasing the *K*_M_ for both NAD^+^ and the acetylated peptide. In addition to its effects on SIRT1, RSV was also shown to act on a wide range of enzymes, including COX-1, cAMP degrading phosphodiesterases, and nuclear factor-κB (NF-κB) (Jang et al., 1997; Manna et al., 2000; Pezzuto, 2011; Park et al., 2012). Consequently, there is debate whether the observed effects of RSV treatment are due to SIRT1 activation. For example, RSV has been shown to promote autophagy and many have attributed this effect to SIRT1 activation (Morselli et al., 2010; Wu et al., 2011). But this assertion was disputed by a study that observed the direct inhibition of mTOR, an inhibitor of autophagy, by RSV (Park et al., 2016). Despite the promiscuous nature of RSV, a study by Price et al. (2012) found that the presence of SIRT1 was necessary for RSV-mediated mitochondrial biogenesis and AMPK activation.

After the discovery of RSV as a SIRT1 activator, the validity of the study and its use of the Fluor de Lys assay was disputed. As the assay uses a synthetic peptide substrate that contains a fluorophore, some argued that it was not physiologically relevant and could produce false positives. Kaeberlein et al. (2005) were able to replicate the activation of SIRT1 by RSV, but only when the fluorescent moiety was present on the peptide substrates. Other studies confirmed that the fluorophore was necessary for RSV-promoted activation of SIRT1 (Borra et al., 2005; Beher et al., 2009; Pacholec et al., 2010). In one such study, Pacholec et al. (2010) used NMR, SPR, and ITC to prove that the sirtuin-activating compounds (STACs) were directly interacting with the fluorophore attached to the peptide, even in the absence of SIRT1. Altogether, the contradictory results called into question the reliability of the fluorometric assay and raised serious doubts concerning RSV’s mechanism of action and its ability to bind to and activate SIRT1 (Schmidt, 2010).

In response to the controversy, scientists at Sirtris, a company established after the initial discovery of SIRT1 activation by RSV, provided an explanation for the importance of the fluorescent moiety. Dai et al. (2010) observed the formation of STAC-substrate complexes, but found no correlation between the potency of a STAC and its affinity for the fluorescent TAMRA group. They further found that the ability of a STAC to activate SIRT1 was dependent on the substrate structure and were able to induce RSV-activation of SIRT1 using substrates composed of natural amino acids (Dai et al., 2010). This finding was also seen by Lakshminarasimhan et al. (2013) in which they observed RSV-activation of SIRT1 after replacing the fluorophore with a large, hydrophobic residue. Hubbard et al. (2013) further found that the fluorophore or its hydrophobic replacement has a positional requirement and were able to show that hydrophobic motifs within PGC-1α and FOXO3a could facilitate SIRT1 activation. Additionally, they also determined that the conserved residue Glu230 was critical for RSV activation and found that the benefits of RSV were attenuated when SIRT1 Glu230 mutants were expressed in myoblasts (Hubbard et al., 2013).

After enough evidence was presented confirming an interaction between RSV and SIRT1, the focus shifted to identifying how RSV interacts with SIRT1. When the association between RSV and SIRT1 was first reported, RSV was categorized as an allosteric modulator of the “K system” type (Monod et al., 1965; Howitz et al., 2003). For “K system” modulators, the *K*_M_ value is affected while the *V*_max_ remains the same. Identifying the region of SIRT1 where RSV binds was the first step in characterizing the interaction. SIRT1 is comprised of three major structured regions: an N-terminal domain (183–229), a catalytic domain (229–516), and a C-terminal regulatory region (641–665) (Davenport et al., 2014). The *apo* enzyme undergoes a conformational change following the binding of NAD^+^ into a “closed” form that traps the substrate (Yuan and Marmorstein, 2012; Davenport et al., 2014). Using hydrogen deuterium exchange mass spectrometry (HDX-MS), Dai et al. (2015) were able to investigate the STAC binding domain (SBD) and determine that residues 183–229 were necessary for STAC activation of SIRT1. A crystal structure of a SIRT1/FdL peptide/RSV complex by Cao et al. (2015) indicated the binding of 3 RSV molecules for each SIRT1. Two of the RSV molecules form hydrogen bonds with the peptide and the SBD, thus bringing the two domains together in a “closed” conformation with improved substrate binding (Cao et al., 2015; Dai et al., 2018). The perspective of RSV acting as a stabilizing force between the substrate and SIRT1 has also been observed in a computational study in which RSV restored binding of “loose-binding” substrates (Hou et al., 2016).

Bioavailability is the primary obstacle in the development of RSV as a therapy. In human trials, Walle et al. (2004) observed that RSV had an absorption of ∼70%, but were unable to detect unmodified RSV 30 min after administration. After absorption, RSV is rapidly metabolized in the liver where it is conjugated to either sulfate or glucuronate (Springer and Moco, 2019). The RSV conjugates are produced by sulfotransferases (SULTs) and uridine-5′-diphospho-glucoronosyltransferases (UGTs) (Springer and Moco, 2019). Both SULTs and UGTs have genetic polymorphisms that can affect their ability to metabolize drugs, and thus can lead to high variability in levels of unmodified RSV between individuals (Ung and Nagar, 2007; Mehboob et al., 2017). The sulfate-conjugated metabolites, which are the primary conjugated form of RSV, were shown to have similar actions to RSV. Calamini et al. (2010) found that the sulfate conjugate of RSV was able to inhibit COX-1 and COX-2, and could activate SIRT1 only in the presence of a Fluor de Lys substrate. As the level of the conjugated forms of RSV surpass that of free RSV, it remains unclear which molecule is responsible for the experimentally observed effects of RSV supplementation.

Development of novel formulations of RSV emerged as its bioavailability became a prominent issue. One approach was to inhibit the enzymes responsible for conjugating RSV. Co-administration of RSV with quercetin was shown to decrease the formation of RSV-sulfate conjugates through the inhibition of SULT1A1, the major SULT isoform expressed in the liver and kidneys (De Santi et al., 2000). Multiple studies have observed the synergistic effect between RSV and quercetin from reducing adipose tissue weight following a high fat diet (Arias et al., 2016) to inhibiting the development of prostate cancer in a mouse model (Singh and Ahmad, 2015). In a similar fashion, Reen et al. (1993) combined RSV with piperine, an alkaloid previously shown to diminish the activity of UGTs in the intestines of rats. The study found that the addition of piperine to RSV resulted in a more than 1000% increase in the maximum serum concentration of RSV (Johnson et al., 2011). Despite the promising results seen in animal models, outcomes from human trials in healthy subjects were ambiguous (Table 1). Two trials co-administering RSV and quercetin found no improvement in RSV pharmacokinetics (la Porte et al., 2010; Huhn et al., 2018). Piperine supplementation was found to be useful in improving RSV bioavailability in one study examining cerebral blood flow (Wightman et al., 2014), but had no beneficial effect on serum levels of RSV in another study (Bailey et al., 2021).

**TABLE 1:** Clinical trials examining pharmacokinetics of novel resveratrol formulations and administrations.

**Formulation/administration**	**Description**	**Outcomes**	**References**
Revifast	Solid dispersion of RSV on Magnesium dihydroxide	Revifast had C_max_ threefold higher than RSV and an earlier peak in RSV	Iannitti et al., 2020
SRT501	Micronized RSV; 5,000 mg/day	SRT501 had a 3.6-fold increase in plasma RSV levels compared to non-micronized RSV	Howells et al., 2011
JOTROL	Micellar emulsion	Recruiting	NCT04668274
With food	High fat meal	RSV taken with food had delayed absorption, but the amount of absorption was not affected	Vaz-da-Silva et al., 2008
RSV/Piperine	2,500 mg RSV + 0/5/25 mg Piperine	No significant change in pharmacokinetics was seen	Bailey et al., 2021
RSV/Quercetin	2,000 mg RSV + 500 mg Quercetin	No significant change in RSV exposure	la Porte et al., 2010

Other groups tried to solve the bioavailability issue by focusing on the drug delivery method. The Fioretti lab developed a novel formulation of RSV as a solid dispersion on Magnesium dihydroxide microparticles. In their early studies, they found that the new formulation (later termed Revifast) was three times more soluble than the unmodified RSV and had enhanced bioavailability in rabbits (Spogli et al., 2018). In human trials, Revifast displayed an earlier peak in RSV as well as a twofold increase in free-RSV levels in plasma (Iannitti et al., 2020). Another method used to increase bioavailability of RSV is micronization. Sirtris developed SRT501, in which they reduced the particle size below 5 μm in order to improve solubility by increasing the surface area of RSV. In an early clinical trial, SRT501 was well tolerated in colorectal cancer (CRC) patients and had an improved C_max_ compared to conventional RSV (Howells et al., 2011). Following that success, SRT501 was used as a treatment for patients with refractory or relapsed multiple myeloma. The clinical trial was terminated early due to severe side effects including nephrotoxicity (Popat et al., 2013). SRT501 was later discontinued for development as the company focused on other SIRT1-activating drug candidates.

**FIGURE 2:**
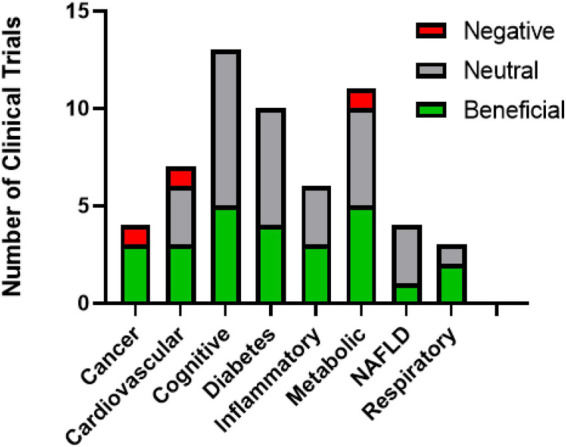
Summary of resveratrol clinical trial outcomes.

RSV has been extensively studied in clinical trials. In the NIH clinical trial registry, over 150 trials using RSV are in various stages of completion. The trials concerned with sirtuin activation and the resulting therapeutic effects have been compiled in Tables 2–9. When summarizing the clinical outcomes of RSV (Figure 2), the issues with RSV are apparent. For most disease states RSV had a neutral effect, suggesting that the bioavailability issues are a major obstacle. Most of the trials in which RSV had a positive effect had higher dosages, typically over 500 mg/day, and the more recent trials are trending toward higher dosages as well. Although it is unclear if RSV can directly activate sirtuins when taken orally, it is likely that work with RSV will continue, as it is a readily available natural product with limited adverse reactions. Despite the early promise seen in the lab, the current reality of RSV has shown the need for developing better, more selective activators of sirtuins.

**TABLE 2:** Resveratrol clinical trials for cancer.

**Condition**	**Phase**	**Subjects**	**Dose**	**Outcome**	**References**
Cancer prevention	I	40	500, 1,000, 2,500, 5,000 mg/day 29 day	RSV safe, but higher doses had GI side effects; RSV treatment caused decrease in IGF-I and IGFBP-3 levels	Brown et al., 2010
CRC	I	20	5,000, 1,000 mg/day 8 day	RSV treatment reduced tumor cell proliferation by 5%	Patel et al., 2010
Cancer prevention	I	42	1,000 mg/day	RSV affected enzymes involved in carcinogen activation and detoxification (CYP3A4, CYP2D6, CYP2C9)	Chow et al., 2010
Multiple Myeloma	II	24	5,000 mg	Terminated early-severe renal side effects	Popat et al., 2013

**TABLE 3:** Resveratrol clinical trials for cardiovascular diseases.

**Condition**	**Phase**	**Subjects**	**Dose**	**Outcome**	**References**
Vascular function	I/II	64	90 mg ResArg	ResArg had improved benefits for vascular function and platelet reactivity compared to RSV	Djurica et al., 2016
Cardiovascular disease	NA	27	300 or 1,000 mg RSV	Higher RSV dosage increased the cardiovascular disease biomarkers, lower RSV dose had no change	Mankowski et al., 2020
Exercise-induced cardiovascular benefits	NA	27	250 mg/day RSV 8 week	RSV diminished positive cardiovascular effects of exercise and had no effect on SIRT1 protein levels	Gliemann et al., 2013
Congestive heart failure	II	40	1,000 mg/day 8 week	Recruiting	NCT03525379
Diabetic coronary artery disease	II	56	500 mg/day 4 week	RSV increased HDL, had beneficial effects on insulin resistance, and upregulated SIRT1	Hoseini et al., 2019
Peripheral artery disease	III	90	125 mg RSV + 1,000 mg NR 6 m	Recruiting	NCT03743636
Peripheral artery disease	NA	66	125 or 500 mg/day	RSV had no consistent effect on walking performance in patients with peripheral artery disease	McDermott et al., 2017
Hypertension	I/II	300	150 or 300 mg/day 12 m	Recruiting	NCT01842399
Endothelial dysfunction	I	24	300 mg	RSV treatment improved endothelial function, but had no effect on blood pressure	Marques et al., 2018
Endothelial dysfunction	NA	25	250 mg	RSV had small beneficial effect on endothelial function, but no additional benefit was seen with exercise	Ozemek et al., 2020

**TABLE 4:** Clinical trials of resveratrol for cognition and neurological disorders.

**Condition**	**Phase**	**Subjects**	**Dose**	**Outcome**	**References**
Alzheimer’s disease	III	27	10 g Dextrose, 10 g Malate, 10 mg RSV/day 12 m	RSV had small, but insignificant effects on mental deterioration	Zhu et al., 2018
Alzheimer’s disease	II	119	500–2,000 mg/day 12 m	RSV and metabolites crossed the BBB; RSV decreased MMP9, neuroinflammation, and induced adaptive immunity	Turner et al., 2015; Moussa et al., 2017
Brain function/structure	NA	60	200 mg RSV, 320 mg Quercetin/day 18 week	No improvement in verbal memory after RSV treatment	Huhn et al., 2018
Cognition and cerebral blood flow	NA	22	250, 500 mg	RSV increased cerebral blood flow, but no change in cognitive function was observed	Kennedy et al., 2010
Cognition	NA	27	500 mg	No cognitive changes seen in healthy patients ages 18–35	Wightman et al., 2019
Cognitive impairment	II/III	40	200 mg/day 26 week	Beneficial, but non-significant changes in markers of diabetes and resting-state functional connectivity	Köbe et al., 2017
Depression	IIII	22	500 mg/day 28 day	RSV did not have a significant antidepressant effect	Aftanas et al., 2020
Friedreich ataxia	I/II	27	1,000 or 5,000 mg/day 12 week	Improvement in oxidative stress markers and ataxia seen only in higher dosage group	Yiu et al., 2013
Friedreich ataxia	II	40	1,000 mg/day Micronized RSV	Recruiting	NCT03933163
Gulf war illness	II	68	2,000 mg/day	Recruiting	NCT03665740
Gulf war illness	NA	64	200–600 mg/day 4 week	RSV reduced Gulf War Illness symptoms	Hodgin et al., 2021
Cognition	I	60	500 mg/day 28 day	RSV treatment reduced fatigue, but had no effect on sleep, health, or cerebral blood flow	Novelle et al., 2015
Cognition	NA	24	500 mg	RSV treatment group had fewer errors in serial subtraction test	Wightman et al., 2019
Schizophrenia	II	19	200 mg/day 4 week	RSV treatment did not improve cognition in patients with schizophrenia	Zortea et al., 2016
Sports concussion	I/II	12	500 mg/day 30 day	No significant effects seen with RSV treatment	NCT01321151

**TABLE 5:** Clinical trials of resveratrol for diabetes.

**Condition**	**Phase**	**Subjects**	**Dose**	**Outcome**	**References**
Dyslipidemia	NA	50	150 mg/day 4 week	RSV treatment did not change cardiovascular or metabolic risk markers	van der Made et al., 2015
Dyslipidemia	II	8	1,000 mg/day, then 2,000 mg/day 2 week	RSV treatment reduced lipoprotein production	Dash et al., 2013
Type 2 diabetes	I	10	3,000 mg/day 12 week	RSV treatment increased SIRT1 and AMPK expression	Goh et al., 2014
Type 2 diabetes	NA	30	2,000–3,000 mg/day 6 week	No changes in T2D markers, but changes in expression of genes involved in mitochondrial activity	Pollack et al., 2017
Type 2 diabetes	NA	17	150 mg/day 30 day	RSV treatment did not improve insulin sensitivity	Timmers et al., 2016
Insulin resistance	NA	112	150 mg/day 12 week	RSV treatment did not impact liver fat content or cardiovascular risk factors	Kantartzis et al., 2018
Type 2 diabetes	NA	54	100 mg/day 2 week then 300 mg/day 2 week	RSV treatment decreased arterial stiffness and had a positive, but insignificant effect on SIRT1 activity	Zhang et al., 2017
Type 2 diabetes	III	192	40 or 500 mg/day 6 m	Higher RSV dosage group had increased SIRT1 levels and antioxidant markers, and decreased H3K56Ac and body fat percentage	Bo et al., 2018
Pre-diabetes	NA	15	150 mg/day 30 day	RSV increased muscle mitochondrial function, but no other metabolic benefits were observed	de Ligt et al., 2018
Pre-diabetes	I	48		Recruiting	NCT02502253
Pre-diabetes	NA	42	150 mg/day 6 m	RSV had no effect on pre-diabetes markers	de Ligt et al., 2020
Insulin resistance	NA	270	RSV + Vitamin C	Recruiting	NCT03090997
Type 2 diabetes	NA	40		Recruiting	NCT03762096
Type 1 diabetes	NA	198		Recruiting	NCT03436992
Type 1 diabetes	Early I	24		Recruiting	NCT04449198

**TABLE 6:** Clinical trials of resveratrol for inflammatory diseases.

**Condition**	**Phase**	**Subjects**	**Dose**	**Outcomes**	**References**
Chronic kidney disease	III	20	500 mg/day 4 week	RSV treatment had no antioxidant or anti-inflammatory effects	Saldanha et al., 2016
Inflammatory markers	NA	44	400 mg RSV + 100 mg Quercetin/day 30 day	RSV treatment had beneficial effect on some inflammatory markers and reduced fasting insulin concentration	Agarwal et al., 2013
Inflammatory markers in smokers	III	40	500 mg/day 30 day	RSV treatment had beneficial effects on some inflammatory markers and the antioxidant level	Bo et al., 2013
Polycystic ovary syndrome	NA	40	1,500 mg/day 3 m	RSV treatment reduced ovarian and adrenal androgens	Banaszewska et al., 2016
Inflammatory markers	NA	22	200 mg RSV + 100 mg Curcumin	RSV/Curcumin treatment had no effect on inflammation after consumption of a high-fat meal	Vors et al., 2018
Endometriosis	IIII	44	40 mg/day 42 day	RSV treatment had no effect on endometriosis pain	Mendes da Silva et al., 2017
Osteoarthritis	III	164		Recruiting	NCT02905799
Chronic kidney disease	NA	25		Recruiting	NCT03597568

**TABLE 7:** Clinical trials of resveratrol for obesity and metabolic disorders.

**Condition**	**Phase**	**Subjects**	**Dose**	**Outcomes**	**References**
Aging	II	60	500 or 1,000 mg/day 12 week	RSV treatment coupled with exercise had beneficial effects on physical and mitochondrial function	Harper et al., 2021
Metabolism	I	32	300 or 1,000 mg/day 90 day	RSV treatment reduced glucose levels in overweight adults	Anton et al., 2014
Metabolic syndrome	NA	25	250 mg/day 3 m	RSV treatment improved many metabolic markers, including total cholesterol, urea, and creatinine	Batista-Jorge et al., 2020
Metabolic syndrome	II	24	1,500 mg/day 90 day	RSV treatment reduced weight, BMI, and total insulin secretion	Méndez-del Villar et al., 2014
Obesity	NA	24	1,500 mg/day 4 week	RSV treatment had no effect	Poulsen et al., 2013
Mitochondrial myopathy	NA	20	1,000 mg/day 8 week	RSV treatment did not improve exercise capacity in subjects with mitochondrial myopathy	Løkken et al., 2019
Obesity	NA	18	150 mg/day 30 day	RSV treatment had no effect on incretin levels, but reduced glucagon levels after eating in obese subjects	Knop et al., 2013
Metabolic syndrome	NA	76	150 or 1,000 mg/day 16 week	RSV treatment did not improve inflammation and increased total cholesterol and LDL cholesterol in subjects with metabolic syndrome	Kjaer et al., 2017
Metabolic syndrome	NA	28	2,000 mg/day 30 day	RSV treatment improvement insulin sensitivity for Caucasian subjects, but non-Caucasian subjects had no difference	Walker et al., 2018
Metabolism	NA	58	75 mg/day 12 week	RSV treatment had no effect on metabolic markers or SIRT1 expression	Yoshino et al., 2012
Obesity	NA	48	500 mg/day 30 day	RSV treatment increased serum levels of SIRT1	Roggerio et al., 2018

**TABLE 8:** Clinical trials of resveratrol for NAFLD.

**Condition**	**Phase**	**Subjects**	**Dose**	**Outcome**	**References**
NAFLD	II/III	50	500 mg/day 12 week	RSV treatment improved inflammatory markers	Faghihzadeh et al., 2014
NAFLD	NA	28	1,500 mg/day 6 m	RSV had no consistent beneficial effect for NAFLD	Heebøll et al., 2016
NAFLD	NA	90	600 mg/day 12 week	RSV treatment led to weight loss, but did not change SIRT1 level or induce other beneficial effects of CR	Asghari et al., 2018
NAFLD	NA	26	1,500 mg/day 6 m	RSV treatment had no effect on metabolic markers for subjects with NAFLD	Poulsen et al., 2018

**TABLE 9:** Clinical trials of resveratrol for respiratory conditions.

**Condition**	**Phase**	**Subjects**	**Dose**	**Outcome**	**References**
Common cold	III	89	Nasal solution of RSV/carboxymethyl-β-glucan	c β G/RSV treatment provided minor benefit for nasal symptoms in infants	Baldassarre et al., 2020
COPD	NA	21	150 mg/day 4 week	RSV treatment did not improve mitochondrial function in subjects with COPD	Beijers et al., 2020
Seasonal allergies	III	76	Nasal solution of RSV/carboxymethyl-β-glucan	c β G/RSV treatment reduced nasal symptoms	Miraglia Del Giudice et al., 2014
COPD	NA	48		Recruiting	NCT03819517
Cystic fibrosis	NA	36		Active, not recruiting	NCT04166396
COVID-19	II	100		Active, not recruiting	NCT04400890
COVID-19	II	60		Active, not recruiting	NCT04542993
COVID-19	NA	30		Recruiting	NCT04799743

### Resveratrol-Related Activators

Following the discovery of RSV, Sirtris began to develop new small molecule activators of SIRT1. From HTS, they discovered SIRT1 activators that were structurally distinct from RSV and had improved SIRT1 activating abilities (Milne et al., 2007). One of the compounds, SRT1720 (Figure 1), activated SIRT1 with an EC_1.5_ = 2.9 μM and a maximum activation of approximately 4.5-fold, whereas RSV had an EC_1.5_ = 46.2 μM and a maximum activation of 2-fold (Milne et al., 2007). Using ITC, it was determined that the SRT STACs could only bind SIRT1 in the presence of the peptide substrate and they had a mechanism of action similar to RSV. It was further found that they used the same binding site as RSV. In a rodent model of insulin resistance, SRT1720 treatment resulted in a decrease in the blood glucose level and an increase in insulin sensitivity (Milne et al., 2007).

Thus far, clinical trials of three compounds related to SRT1720 have been completed. SRT2104 (Figure 1) has the greatest number of registered clinical trials (Table 10), but SRT2379 and SRT3025 (Figure 1) have also made it into the clinic (Table 11). The single clinical trial to assess the safety and pharmacokinetics of SRT3025 was interrupted after the researchers found a prolongation effect of SRT3025 on the corrected QT interval, a warning that continuation could lead to a potentially fatal proarrhythmia in the subjects (NCT01340911, GSK Study Register). Further development of SRT2379 was also terminated due to preclinical toxicities observed by the researchers (NCT01416376, GSK Study Register).

**TABLE 10:** Clinical trials on SRT2104.

**Condition**	**Phase**	**Subjects**	**Dose**	**Outcome**	**References**
Pharmacokinetics	I	20	500 mg	SRT2104 had increased absorption with ingested with food; Headache was most common AE	Hoffmann et al., 2013
Type 2 Diabetes	I	10	2,000 mg/day 7 day	SRT2104 increased after multiple doses; Headache was the most common AE (affecting 50% of the treatment group)	Hoffmann et al., 2013
COPD	I	20	250–2,000 mg	SRT2104 had no effect on inflammatory markers; variable pharmacokinetic parameters	NCT00920660, GSK Study Register
Muscular atrophy	I	24	500 or 2,000 mg/day 28 day	SRT2104 treatment decreased cholesterol and LDL levels, but had variable pharmacokinetics.	Libri et al., 2012
Sepsis/Inflammation	I	24	2,000 mg/day 7 day	SRT2104 treatment had anti-inflammatory and anticoagulant effects	van der Meer et al., 2015
Type 2 diabetes	I	38	2,000 mg/day 28 day	SRT2104 treatment had a beneficial metabolic effect and improved lipid profiles and arterial stiffness. It had inconsistent effects on endothelial function	Venkatasubramanian et al., 2013, 2016; Noh et al., 2017
Psoriasis	II	40	250, 500, 1,000 mg/day 84 day	35% of SRT2104 treatment group had improvement in psoriasis; 69% had AEs; SRT2104 exposure was highly variable	Krueger et al., 2015
Type 2 diabetes	II	86	2,000 mg/day 28 day	SRT2104 had no consistent effects on insulin sensitivity	NCT01018017, GSK Study Register
Type 2 diabetes	I	227	250–2,000 mg/day 28 day	SRT2104 did not improve glucose or insulin control; Exposure was highly variable	Baksi et al., 2014
Ulcerative colitis	II	17	50,500 mg/day 8 week	SRT2104 did not improve UC	Sands et al., 2016
Pharmacokinetics	I	65	30–3,000 mg/day 7 day	SRT2104 bioavailability was 14%; Administration with food increased drug exposure	Hoffmann et al., 2013

**TABLE 11:** Clinical trials of SRT2379 and SRT3025.

**Condition**	**Phase**	**Subjects**	**Dose**	**Outcome**	**References**
Type 2 diabetes	I	64	25–3,000 mg SRT2379	SRT2379 exposure increased in a dose-dependent manner	NCT01018628, GSK Study Register
Inflammation	I	17	1,000 mg SRT2379	SRT2379 treatment had a trend of anti-inflammatory effects, but was not statistically significant	NCT01262911, GSK Study Register
Inflammation	I	39	50–1,000 mg SRT2379	SRT2379 treatment did have a significant anti-inflammatory effect	Wiewel et al., 2013
Type 2 diabetes	I	78	50–3,000 mg SRT3025	SRT3025 treatment stopped due to potential adverse cardiovascular side effects	NCT01340911, GSK Study Register

For the lead compound, SRT2104, 5 out of the 8 clinical trials that focused on clinical outcomes had neutral or statistically insignificant results. A pharmacokinetic study found that the bioavailability of SRT2104 was 14% and exposure was improved when administered with food (Hoffmann et al., 2013). Most trials observed highly variable pharmacokinetics, leading some to have inconsistent clinical outcomes. A few trials observed beneficial effects of SRT2104 treatment on lipid profiles (Baksi et al., 2014), histological examinations of subjects with psoriasis (Krueger et al., 2015), and inflammation (van der Meer et al., 2015). McCallum et al. (2014) tried to improve the pharmacokinetics of SRT2104 by using different release formulations, but were unsuccessful. As of current, it appears that SRT2104 is no longer in development. Despite issues observed in clinical trials, SRT2104 continues to be used in studies as a SIRT1 activator (Miller et al., 2021).

## EX-527 (Selisistat)

**FIGURE 3:**
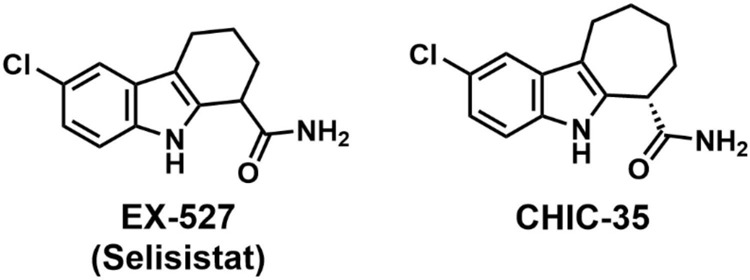
Chemical structures of EX-527 and CHIC-35.

Before 2005, many small molecule regulators targeting sirtuins showed modest potency, isoform selectivity, and solubility (Bedalov et al., 2001; Grozinger et al., 2001; Bitterman et al., 2002; Mai et al., 2005; Olaharski et al., 2005; Porcu and Chiarugi, 2005). Isoform-selective small molecule probes were highly sought after for a better understanding of the biological functions of these enzymes (Porcu and Chiarugi, 2005). Napper et al. (2005) performed an HTS of a library of 280,000 compounds against human recombinant SIRT1 using a fluorometric assay in order to expand available SIRT1 small-molecule probes. The initial screening identified indole as a viable scaffold for SIRT1 targeting. The subsequent SAR study led to the discovery of EX-527 (or Selisistat) and the structurally related compound CHIC-35 (Figure 3) as selective SIRT1 inhibitors with IC_50_ values of 0.098 and 0.063 μM, respectively (Napper et al., 2005). EX-527 exhibited 100-fold selectivity for SIRT1 over SIRT2/3, Class I/II HDACs, and NAD^+^ glycohydrolase (Napper et al., 2005). Kinetic studies indicated that EX-527 is an uncompetitive inhibitor of SIRT1 regarding NAD^+^; thus, inhibition depends on the concentration of NAD^+^ (Napper et al., 2005; Gertz et al., 2013). Additionally, EX-527 exhibits exceptional ADME properties such as oral bioavailability, metabolic stability, and membrane penetrability, thus elevating EX-527 from a small-molecule probe to a therapeutic candidate (Napper et al., 2005). In contrast, the study of CHIC-35 has been limited to pre-clinical investigations, primarily on its anti-inflammatory effects (Lugrin et al., 2013; Asad and Sachidanandan, 2020).

SAR studies of EX-527 revealed that the primary carboxamide was necessary for an effective inhibition and modifications to the lead compound were generally not well tolerated (Napper et al., 2005). The isoform selectivity of EX-527 was initially attributed to possible differences among the sirtuin active sites (Gertz et al., 2013; Broussy et al., 2020). A recent structural biology study suggested that EX-527 occupies the C-pocket (nicotinamide binding site) and a neighboring hydrophobic pocket which are highly conserved in the sirtuin family (Gertz et al., 2013). Further kinetic analysis indicated that the isoform selectivity of EX-527 stemmed from the differences in the kinetics of catalysis rather than any significant structural variation (Gertz et al., 2013; Broussy et al., 2020).

EX-527 has been explored as a potential therapeutic for Huntington’s disease (HD). HD is a neurodegenerative disease characterized by abnormal movements, personality changes, and cognitive decline (Bates et al., 2015). A hallmark of HD is the expression of the mutant Huntington gene (m*HTT*), which has an expansion of a cytosine-adenine-guanine (CAG) repeat (Bates et al., 2015; Tabrizi et al., 2020). This extension leads to the protein misfolding and the formation of protein aggregates in HD patients (Bates et al., 2015; Tabrizi et al., 2020). As such, a potential therapeutic approach has focused on the degradation and removal of these aggregates. Acetylation of mHTT can direct the protein to autophagosomes for degradation (Jeong et al., 2009), thus facilitating the removal of the mutant protein. SIRT1 has been pursued as a therapeutic target for the treatment of HD because it has been shown to deacetylate mHTT to prevent its degradation. Genetic loss or pharmacological inhibition of Sir2 (the *Drosophila melanogaster* homolog of human SIRT1) was found to be neuroprotective for m*HTT*-challenged fruit flies (Pallos et al., 2008). Indeed, Smith et al. (2014) demonstrated that in a *Drosophila* model of HD, 10 μM of EX-527 could rescue neurodegeneration at a comparable level as the genetic elimination of Sir2. Additionally, in the R6/2 mouse model of HD, EX-527 can restore neural functions at 5 and 20 mg/kg dosages (Smith et al., 2014). It is important to note that the role of SIRT1 in HD remains controversial, as some view it as protective in HD modulation (Duan, 2013).

The aforementioned preclinical animal studies were essential for clinical trials involving EX-527 as an HD treatment option (Table 12). In a Phase 1 trial (NCT01521832), EX-527 was assessed for its safety in healthy human subjects. The study employed healthy men and women with two separate dosage regimens: a single dosage of 5, 25, 75, 150, 300, or 600 mg, and multiple dosages of 100, 200, or 300 mg/day (Westerberg et al., 2015). In this study, EX-527 was found to be well-tolerated and safe after multiple doses of 300 mg/day and at a single dose of 600 mg. Based on the promising safety profile and dosing information, another clinical trial (NCT01485952) sought to investigate the feasibility of targeting SIRT1 as a potential treatment for HD (Sussmuth et al., 2015). In this study, human subjects with HD were treated with either 0, 10, or 100 mg/day of EX-527. There were improvements across the clinical, cognitive, and neuropsychiatric assessments from the baseline (day -1) to day 1 with no additional improvement at day 14 (Sussmuth et al., 2015).

**TABLE 12:** Clinical trials of EX-527 for Huntington’s disease.

**Condition**	**Phase**	**Subjects**	**Dose**	**Outcome**	**References**
Huntington’s disease	I	55	10, 100 mg/day 14 day	EX-527 was well tolerated in early stage HD patients at 10 and 100 mg/day, baseline to day 1 improvement	Sussmuth et al., 2015
Huntington’s disease	I	26	100 mg/day 14 day	N.R.P^a^	NCT01485965
Healthy subjects	I	88	5, 25, 75, 150, 300, 600 mg/day 100, 300 mg/day	EX-527 was well tolerated at a max single dose of 600 mg and max multiple doses of 300 mg/day	Westerberg et al., 2015
Huntington’s disease	II	144	50, 200 mg/day	N.R.P	NCT01521585

*^a^N.R.P, no results posted.*

A relatively new clinical application of EX-527 is improving *in vitro* fertilization (IVF) outcomes through the treatment of endometriosis (NCT04184323). Endometriosis is a chronic inflammatory reproductive disorder involving the growth of uterine endometrial cells outside of the uterine cavity (Zondervan et al., 2020). These delocalized endometrial growths can become lesions that lead to chronic localized pelvic pain and cramping with accompanying fertility issues. Standard treatment options for endometriosis involve mechanical lesion removal, hormonal therapy, or a combination of the two (Budinetz and Sanfilippo, 2010). However, reemergence of the lesions and complications associated with the hormonal therapy remain limitations. Thus, alternative treatment options are needed. A recent study demonstrated a KRAS activation-triggered SIRT1 overexpression in women with endometriosis, which has been suggested to contribute to infertility and the pathogenesis of endometriosis (Yoo et al., 2017). Targeting SIRT1 with small molecule inhibitors thus serves as a potential therapeutic treatment for endometriosis-mediated IVF failure. A planned clinical trial (NCT04184323) will seek to explore inhibition of SIRT1 by EX-527 as a possible treatment for the inflammation associated with endometriosis.

## Quercetin

**FIGURE 4:**
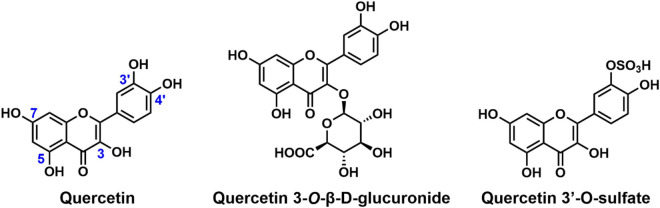
Chemical structures of quercetin and its derivatives.

Quercetin (Figure 4) is a flavonoid phytoestrogen which has demonstrated activity in the management of brain, blood, salivary gland and uterine cancers (Dolatabadi, 2011; Sak, 2014; Sinaga et al., 2017), as well as viral infections such as HCV (Rojas et al., 2016) and Zika virus (Wong et al., 2017) and bacterial infections (Wang et al., 2018; Zeng et al., 2019; Oktyabrsky et al., 2020) in both *in vivo* and *in vitro* studies. The structure of quercetin consists of three rings and five hydroxyl groups at the 3, 5, 7, 3′ and 4′-positions of the basic flavanol skeleton (Figure 4). The name “quercetin” was derived from the Latin word “Quercetum” which means oak forest. It is one of the most abundant flavonoids found in fruits and vegetables, and was discovered alongside other bioflavonoids by Albert Szent Gyorgyi in 1936 (Moskaug, Carlsen et al., 2004).

In the same HTS that identified RSV, quercetin was observed to increase SIRT1 activity by fivefold (Howitz et al., 2003). Studies have shown the inhibitory role of quercetin on the progression of breast, colon, prostate, and lung cancers (Baghel et al., 2012; Smith et al., 2016). Quercetin has been found to alleviate kidney fibrosis, intervertebral disc degeneration, and diabetic encephalopathy *via* activation of SIRT1-mediated pathways (Dong et al., 2014; Hu et al., 2020; Liu et al., 2020). Quercetin treatment of Herpes simplex virus-1 infected neuronal cell lines increased the survival of the cells by inhibiting viral production and improved neurodegenerative markers *via* SIRT1 activation (Leyton et al., 2015). It was also found that quercetin inhibits oxidative injury in human endothelial cells through SIRT1 activation, leading to the upregulation of the SIRT1/AMPK pathway (Chen et al., 2013). In addition, quercetin regulates oxidative stress in the body by directly reducing the level of reactive oxygen species (Oboh et al., 2016). In myocardial/ischemia-reperfusion (MI/R) injury in rats, quercetin supplementation increased the expression levels of SIRT1 and PGC-1α, leading to the activation of the SIRT1/PGC-1α pathway and subsequent reduction in MI/R-induced myocardial damage (Tang et al., 2019). In addition to SIRT1, quercetin has a mild stimulating effect on SIRT6 (You et al., 2019). When modified with a bulky trihydroxy benzoyl group at the 3-OH group, as in catechin gallate, it inhibits SIRT6 activity (Rahnasto-Rilla et al., 2018; You et al., 2019).

Quercetin exists as a glycone or an aglycone in plants. When ingested, the glycone form can be hydrolyzed to the aglycone form that can be easily absorbed in the small intestine due to its hydrophobic nature (Massi et al., 2017). In human plasma, ingested quercetin glycosides are predominantly metabolized into quercetin 3-*O*-β-D-glucuronide and quercetin 3′-*O*-sulfate (Figure 4; D’Andrea, 2015; Moodi et al., 2021). Modifications such as glycosylation and methylation of the quercetin scaffold result in derivatives with distinct biological activities (Lesjak et al., 2018). For example, isoquercetin, the 3-*O*-glucoside of quercetin (Magar and Sohng, 2020), demonstrates SIRT6 stimulation activity with no influence on SIRT1 activity (You et al., 2019).

Quercetin’s therapeutic applications have been limited by its low bioavailability, poor solubility, and short half-life (Gugler et al., 1975; Ferry et al., 1996; KaŞıkcı and Bağdatlıoğlu, 2016). Modifications have been made to improve these properties (Massi et al., 2017). In one study, the bioavailability of quercetin was increased by about 20 times through a phytosome delivery system (Riva et al., 2019). Several other studies have utilized conjugation to various amino acids and nanoparticle delivery systems to improve the bioavailability of quercetin. Acylated quercetin analogs synthesized by Duan et al. (2017) were about 10-fold more soluble in water than quercetin. Of the several quercetin human clinical trials targeting various disease states, only one ongoing study (NCT03943459) is aimed at investigating the activation of SIRT1 by quercetin in coronary disease.

## Conclusion and Perspectives

A simple search in PubMed provides hundreds of publications related to sirtuin inhibitor/activator development, demonstrating the critical roles these enzymes play in regulating diverse cellular events and the intense interest in pursuing them as therapeutic targets. Unfortunately, tremendous efforts have only resulted in a handful of small molecules in clinical studies as described in this review article. Translating sirtuin regulators from the bench to the clinics has been hampered by the lack of isoform-selective candidate compounds with favorable pharmacological profiles. The catalytic domain is highly conserved between sirtuins and therefore represents a promiscuous target for NAD^+^ or peptide-competitive inhibitors (Dai et al., 2018). In the case of activators, the binding sites are often not readily apparent by the inspection of a crystal structure, and there is no general and systematic strategy to identify and target these sites. Furthermore, for several human sirtuin isoforms, novel enzymatic activities were discovered recently (Du et al., 2011; Feldman et al., 2013; Jiang et al., 2013; Anderson et al., 2017). Potent regulators targeting specifically these new activities are still in the making. Pre-clinical investigations using animal models may differ in the genetic background or the assessment methods which have caused controversies and ambiguities that still need to be reconciled. In spite of numerous reports on the endogenous substrates of sirtuins and the pathways they regulate, our understanding of the biological functions of sirtuins is still in its infancy. For example, SIRT1 has been closely associated with cancer pathology, and has been suggested as either a tumor promoter or a suppressor in a context-dependent manner (Deng, 2009). All the research effort has only scratched the surface of sirtuin biology. A comprehensive and thorough picture of these intriguing enzymes still awaits description.

Of course, sirtuin-targeting drugs still hold great therapeutic potential, and progress in the field will accelerate the development of small molecule drug candidates. Apart from their highly conserved catalytic core, sirtuins harbor structurally unique N- or C-terminal extensions that can be targeted for selectivity or even specificity. The conformational plasticity of the active site that explains the isoform selectivity of EX-527 (Gertz et al., 2013; Broussy et al., 2020) has also been suggested as a novel target for inhibitor development. The clinical success of sirtuin-targeting medications requires a clear understanding of the “sirtuin-dependency” of the disease, robust lead compounds that are potent and selective with ideal drug-like properties, PK/PD profiling and improvement, as well as advances in formulation. The combined efforts from all these aspects will bring more sirtuin regulators into the clinic for treating diseases with considerable unmet medical needs.

## Author Contributions

AC, DW, and YC: conceptualization. AC, DW, DD, and YC: writing. YC: project administration and funding acquisition. All authors contributed to the article and approved the submitted version.

## Conflict of Interest

The authors declare that the research was conducted in the absence of any commercial or financial relationships that could be construed as a potential conflict of interest.

## Publisher’s Note

All claims expressed in this article are solely those of the authors and do not necessarily represent those of their affiliated organizations, or those of the publisher, the editors and the reviewers. Any product that may be evaluated in this article, or claim that may be made by its manufacturer, is not guaranteed or endorsed by the publisher.

## Abbreviations

A β: Amyloid beta
AD: Alzheimer’s disease
ADME: Absorptiondistribution, metabolism excretion
AE: Adverse effect
AMPK: AMP-activated protein kinase
BBB: Blood-brain barrier
BDNF: Brain-derived neurotrophic factor
C_max_: Maximum concentration of a drug in the intended organ/tissue after administration
CML: Chronic myeloid leukemia
COPD: Chronic obstructive pulmonary disease
COX: Cyclooxygenase
CRC: Colorectal cancer
EC_1.5_: Concentration at which the enzyme activity is increased by 50%
FOXO: Forkhead box transcription factor
GI: gastrointestinal
HD: Huntington’s disease
HDACS: Histone deacetylases
HDL: High density lipoprotein
HDX-MS: Hydrogen deuterium exchange mass spectrometry
HTS: High-throughput screening
IC_50_: Half-maximal inhibitory concentration
IGFBP: Insulin-like growth factor binding protein
IGF-I: Insulin-like growth factor I
IHD: Ischemic heart disease
ITC: Isothermal colorimetry
*K*_m_: Michaelis-Menten constant
LDL: Low density lipoprotein
m*HTT*: mutant Huntington gene
MI/R- Myocardial/ischemia-reperfusion, MOA: Mechanism of action
NAD: Nicotinamide adenine dinucleotide
NAFLD: Non-alcoholic fatty liver disease
NF- κ B: Nuclear factor κ B
NFTs: Neurofibrillary tangles
NMR: Nuclear magnetic resonance
PD: Pharmacodynamic
PGC: Peroxisome proliferator-activated receptor-gamma coactivator
PK: Pharmacokinetic
PTEN: Phosphatase and tensin homolog
RSV: Resveratrol
SAR: Structure-activity relationship
SBD: STAC binding domain
SPR: Surface plasmon resonance
STACs: Sirtuin-activating compounds
SULTs: Sulfotransferases
TAMRA: Tetramethylrhodamine
T2D: Type 2 diabetes mellitus
UC: Ulcerative colitis
UGTs: Uuridine-5′-diphospho-glucoronosyltransferases.

## References

[B1] Aftanas L. I. Markov A. A. Rikita M. V. Danilenko K. V. (2020). P.326 Efficacy of resveratrol in the treatment of unipolar depression: double-blind randomized placebo-controlled parallel-group study. *Eur. Neuropsychopharmacol.* 40:S189.

[B2] Agarwal B. Campen M. J. Channell M. M. Wherry S. J. Varamini B. Davis J. G. (2013). Resveratrol for primary prevention of atherosclerosis: clinical trial evidence for improved gene expression in vascular endothelium. *Int. J. Cardiol.* 166 246–248. 10.1016/j.ijcard.2012.09.027 23098852 PMC3646959

[B3] Anderson K. A. Huynh F. K. Fisher-Wellman K. Stuart J. D. Peterson B. S. Douros J. D. (2017). SIRT4 is a lysine deacylase that controls leucine metabolism and insulin secretion. *Cell Metab.* 25 838–855.e15. 10.1016/j.cmet.2017.03.003 28380376 PMC5444661

[B4] Anton S. D. Embry C. Marsiske M. Lu X. Doss H. Leeuwenburgh C. (2014). Safety and metabolic outcomes of resveratrol supplementation in older adults: results of a twelve-week, placebo-controlled pilot study. *Exp. Gerontol.* 57 181–187. 10.1016/j.exger.2014.05.015 24866496 PMC4149922

[B5] Arias N. Macarulla M. T. Aguirre L. Milton I. Portillo M. P. (2016). The combination of resveratrol and quercetin enhances the individual effects of these molecules on triacylglycerol metabolism in white adipose tissue. *Eur. J. Nutr.* 55 341–348. 10.1007/s00394-015-0854-9 25669932

[B6] Asad Z. Sachidanandan C. (2020). Chemical screens in a zebrafish model of CHARGE syndrome identifies small molecules that ameliorate disease-like phenotypes in embryo. *Eur. J. Med. Genet.* 63:103661. 10.1016/j.ejmg.2019.04.018 31051269

[B7] Asghari S. Asghari-Jafarabadi M. Somi M.-H. Ghavami S.-M. Rafraf M. (2018). Comparison of calorie-restricted diet and resveratrol supplementation on anthropometric indices, metabolic parameters, and serum Sirtuin-1 levels in patients with nonalcoholic fatty liver disease: a randomized controlled clinical trial. *J. Am. Coll. Nutr.* 37 223–233. 10.1080/07315724.2017.1392264 29313746

[B8] Baghel S. S. Shrivastava N. Baghel R. S. Agrawal P. Rajput S. (2012). A review of quercetin: antioxidant and anticancer properties. *World J. Pharm. Pharm. Sci.* 1 146–160.

[B9] Bailey H. H. Johnson J. J. Lozar T. Scarlett C. O. Wollmer B. W. Kim K. (2021). A randomized, double-blind, dose-ranging, pilot trial of piperine with resveratrol on the effects on serum levels of resveratrol. *Eur. J. Cancer Prev.* 30 285–290. 32868637 10.1097/CEJ.0000000000000621 PMC7910313

[B10] Baksi A. Kraydashenko O. Zalevkaya A. Stets R. Elliott P. Haddad J. (2014). A phase II, randomized, placebo-controlled, double-blind, multi-dose study of SRT2104, a SIRT1 activator, in subjects with type 2 diabetes. *Br. J. Clin. Pharmacol.* 78 69–77. 10.1111/bcp.12327 24446723 PMC4168381

[B11] Baldassarre M. E. Di Mauro A. Labellarte G. Pignatelli M. Fanelli M. Schiavi E. Mastromarino P. Capozza M. Panza R. Laforgia N. (2020). Resveratrol plus carboxymethyl-β-glucan in infants with common cold: a randomized double-blind trial. *Heliyon* 6:e03814. 10.1016/j.heliyon.2020.e03814 32322697 PMC7172624

[B12] Banaszewska B. Wrotyńska-Barczyńska J. Spaczynski R. Z. Pawelczyk L. Duleba A. J. (2016). Effects of resveratrol on polycystic ovary syndrome: a double-blind, randomized, placebo-controlled trial. *J. Clin. Endocrinol. Metab.* 101 4322–4328. 10.1210/jc.2016-1858 27754722

[B13] Bates G. P. Dorsey R. Gusella J. F. Hayden M. R. Kay C. Leavitt B. R. (2015). Huntington disease. *Nat. Rev. Dis. Primers* 1:15005. 27188817 10.1038/nrdp.2015.5

[B14] Batista-Jorge G. C. Barcala-Jorge A. S. Silveira M. F. Lelis D. F. Andrade J. M. O. de Paula A. M. B. (2020). Oral resveratrol supplementation improves Metabolic Syndrome features in obese patients submitted to a lifestyle-changing program. *Life Sci.* 256:117962. 10.1016/j.lfs.2020.117962 32534040

[B15] Bedalov A. Gatbonton T. Irvine W. P. Gottschling D. E. Simon J. A. (2001). Identification of a small molecule inhibitor of Sir2p. *Proc. Natl. Acad. Sci. U.S.A*. 98 15113–15118. 11752457 10.1073/pnas.261574398 PMC64992

[B16] Beher D. Wu J. Cumine S. Kim K. W. Lu S.-C. Atangan L. (2009). Resveratrol is not a direct activator of SIRT1 enzyme activity. *Chem. Biol. Drug Des.* 74 619–624. 19843076 10.1111/j.1747-0285.2009.00901.x

[B17] Beijers R. J. Gosker H. R. Sanders K. J. de Theije C. Kelders M. Clarke G. (2020). Resveratrol and metabolic health in COPD: a proof-of-concept randomized controlled trial. *Clin. Nutr.* 39 2989–2997. 10.1016/j.clnu.2020.01.002 31996311

[B18] Bertelli A. A. Giovannini L. Giannessi D. Migliori M. Bernini W. Fregoni M. (1995). Antiplatelet activity of synthetic and natural resveratrol in red wine. *Int. J. Tissue React.* 17 1–3. 7499059

[B19] Bitterman K. J. Anderson R. M. Cohen H. Y. Latorre-Esteves M. Sinclair D. A. (2002). Inhibition of silencing and accelerated aging by nicotinamide, a putative negative regulator of yeast sir2 and human SIRT1. *J. Biol. Chem*. 277 45099–45107. 10.1074/jbc.M205670200 12297502

[B20] Blander G. Guarente L. (2004). The Sir2 family of protein deacetylases. *Annu. Rev. Biochem*. 73 417–435. 15189148 10.1146/annurev.biochem.73.011303.073651

[B21] Bo S. Ciccone G. Castiglione A. Gambino R. De Michieli F. Villois P. (2013). Anti-inflammatory and antioxidant effects of resveratrol in healthy smokers a randomized, double-blind, placebo-controlled, cross-over trial. *Curr. Med. Chem.* 20 1323–1331. 10.2174/0929867311320100009 23298135

[B22] Bo S. Togliatto G. Gambino R. Ponzo V. Lombardo G. Rosato R. (2018). Impact of sirtuin-1 expression on H3K56 acetylation and oxidative stress: a double-blind randomized controlled trial with resveratrol supplementation. *Acta Diabetol.* 55 331–340. 10.1007/s00592-017-1097-4 29330620 PMC5851693

[B23] Borra M. T. Smith B. C. Denu J. M. (2005). Mechanism of human SIRT1 activation by resveratrol. *J. Biol. Chem.* 280 17187–17195. 15749705 10.1074/jbc.M501250200

[B24] Broussy S. Laaroussi H. Vidal M. (2020). Biochemical mechanism and biological effects of the inhibition of silent information regulator 1 (SIRT1) by EX-527 (SEN0014196 or selisistat). *J. Enzyme Inhib. Med. Chem.* 35 1124–1136. 10.1080/14756366.2020.1758691 32366137 PMC7241506

[B25] Brown V. A. Patel K. R. Viskaduraki M. Crowell J. A. Perloff M. Booth T. D. (2010). Repeat dose study of the cancer chemopreventive agent resveratrol in healthy volunteers: safety, pharmacokinetics, and effect on the insulin-like growth factor axis. *Cancer Res.* 70 9003–9011. 10.1158/0008-5472.CAN-10-2364 20935227 PMC2982884

[B26] Budinetz T. Sanfilippo J. S. (2010). Treatment of endometriosis: a hormonal approach. *Minerva Ginecol*. 62 373–380. 20827253

[B27] Burr M. L. (1995). Explaining the French paradox. *J. R. Soc. Health* 115 217–219. 7562866 10.1177/146642409511500404

[B28] Calamini B. Ratia K. Malkowski M. G. Cuendet M. Pezzuto J. M. Santarsiero B. D. (2010). Pleiotropic mechanisms facilitated by resveratrol and its metabolites. *Biochem. J.* 429 273–282. 10.1042/BJ20091857 20450491 PMC3265359

[B29] Cao D. Wang M. Qiu X. Liu D. Jiang H. Yang N. (2015). Structural basis for allosteric, substrate-dependent stimulation of SIRT1 activity by resveratrol. *Genes Dev*. 29 1316–1325. 10.1101/gad.265462.115 26109052 PMC4495401

[B30] Cen Y. Youn D. Y. Sauve A. A. (2011). Advances in characterization of human sirtuin isoforms: chemistries, targets and therapeutic applications. *Curr. Med. Chem.* 18 1919–1935. 21517779 10.2174/092986711795590084

[B31] Chang A. R. Ferrer C. M. Mostoslavsky R. (2020). SIRT6, a Mammalian Deacylase with Multitasking Abilities. *Physiol. Rev.* 100 145–169. 10.1152/physrev.00030.2018 31437090 PMC7002868

[B32] Chang H. C. Guarente L. (2014). SIRT1 and other sirtuins in metabolism. *Trends Endocrinol. Metab.* 25 138–145. 24388149 10.1016/j.tem.2013.12.001 PMC3943707

[B33] Chen Z. Shentu T.-P. Wen L. Johnson D. A. Shyy J. Y. J. (2013). Regulation of SIRT1 by oxidative stress-responsive miRNAs and a systematic approach to identify its role in the endothelium. *Antioxid. Redox Signal.* 19 1522–1538. 10.1089/ars.2012.4803 23477488 PMC3797452

[B34] Chow H. H. S. Garland L. L. Hsu C.-H. Vining D. R. Chew W. M. Miller J. A. (2010). Resveratrol modulates drug- and carcinogen-metabolizing enzymes in a healthy volunteer study. *Cancer Prev. Res*. 3:1168. 10.1158/1940-6207.CAPR-09-0155 20716633 PMC2933312

[B35] Dai H. Case A. W. Riera T. V. Considine T. Lee J. E. Hamuro Y. (2015). Crystallographic structure of a small molecule SIRT1 activator-enzyme complex. *Nat. Commun.* 6:7645. 10.1038/ncomms8645 26134520 PMC4506539

[B36] Dai H. Kustigian L. Carney D. Case A. Considine T. Hubbard B. P. (2010). SIRT1 activation by small molecules: kinetic and biophysical evidence for direct interaction of enzyme and activator. *J. Biol. Chem.* 285 32695–32703. 10.1074/jbc.M110.133892 20702418 PMC2963390

[B37] Dai H. Sinclair D. A. Ellis J. L. Steegborn C. (2018). Sirtuin activators and inhibitors: Promises, achievements, and challenges. *Pharmacol. Ther*. 188 140–154. 10.1016/j.pharmthera.2018.03.004 29577959 PMC6342514

[B38] D’Andrea G. (2015). Quercetin: a flavonol with multifaceted therapeutic applications? *Fitoterapia* 106 256–271. 10.1016/j.fitote.2015.09.018 26393898

[B39] Dash S. Xiao C. Morgantini C. Szeto L. Lewis G. F. (2013). High-dose resveratrol treatment for 2 weeks inhibits intestinal and hepatic lipoprotein production in overweight/obese men. *Arterioscler. Thromb. Vasc. Biol.* 33 2895–2901. 10.1161/ATVBAHA.113.302342 24072699

[B40] Davenport A. M. Huber F. M. Hoelz A. (2014). Structural and functional analysis of human SIRT1. *J. Mol. Biol.* 426 526–541. 24120939 10.1016/j.jmb.2013.10.009 PMC4211926

[B41] de Ligt M. Bergman M. Fuentes R. M. Essers H. Moonen-Kornips E. Havekes B. (2020). No effect of resveratrol supplementation after 6 months on insulin sensitivity in overweight adults: a randomized trial. *Am. J. Clin. Nutr.* 112 1029–1038. 10.1093/ajcn/nqaa125 32492138 PMC7528554

[B42] de Ligt M. Bruls Y. M. H. Hansen J. Habets M. F. Havekes B. Nascimento E. B. M. (2018). Resveratrol improves *ex vivo* mitochondrial function but does not affect insulin sensitivity or brown adipose tissue in first degree relatives of patients with type 2 diabetes. *Mol. Metab.* 12 39–47. 29706321 10.1016/j.molmet.2018.04.004 PMC6001939

[B43] De Santi C. Pietrabissa A. Spisni R. Mosca F. Pacifici G. M. (2000). Sulphation of resveratrol, a natural compound present in wine, and its inhibition by natural flavonoids. *Xenobiotica* 30 857–866. 11055264 10.1080/004982500433282

[B44] Deng C. X. (2009). SIRT1, is it a tumor promoter or tumor suppressor? *Int. J. Biol. Sci.* 5 147–152. 19173036 10.7150/ijbs.5.147 PMC2631220

[B45] Djurica D. Ren J. Holt R. R. Feng X. Carlson C. R. Shindel A. W. (2016). A single intake of a resveratrol-arginine conjugate improves microvascular function compared to trans-resveratrol in postmenopausal women. *Pharma Nutr.* 4 132–138.

[B46] Dolatabadi J. E. N. (2011). Molecular aspects on the interaction of quercetin and its metal complexes with DNA. *Int. J. Biol. Macromol.* 48 227–233. 10.1016/j.ijbiomac.2010.11.012 21115036

[B47] Dong J. Zhang X. Zhang L. Bian H. X. Xu N. Bao B. Liu J. (2014). Quercetin reduces obesity-associated ATM infiltration and inflammation in mice: a mechanism including AMPKalpha1/SIRT1. *J. Lipid Res.* 55 363–374. 10.1194/jlr.M038786 24465016 PMC3934722

[B48] Du J. Zhou Y. Su X. Yu J. J. Khan S. Jiang H. (2011). Sirt5 is a NAD-dependent protein lysine demalonylase and desuccinylase. *Science* 334 806–809. 22076378 10.1126/science.1207861 PMC3217313

[B49] Duan W. (2013). Targeting sirtuin-1 in Huntington’s disease: rationale and current status. *CNS Drugs* 27 345–352. 10.1007/s40263-013-0055-0 23549885 PMC3660428

[B50] Duan Y. Sun N. Xue M. Wang X. Yang H. (2017). Synthesis of regioselectively acylated quercetin analogues with improved antiplatelet activity. *Mol. Med. Rep.* 16 9735–9740. 10.3892/mmr.2017.7781 29039540

[B51] Endres K. Fahrenholz F. (2012). The role of the anti-amyloidogenic secretase ADAM10 in shedding the app-like proteins. *Curr. Alzheimer Res*. 9 157–164. 10.2174/156720512799361664 21605036

[B52] Faghihzadeh F. Adibi P. Rafiei R. Hekmatdoost A. (2014). Resveratrol supplementation improves inflammatory biomarkers in patients with nonalcoholic fatty liver disease. *Nutr. Res.* 34 837–843. 10.1016/j.nutres.2014.09.005 25311610

[B53] Feldman J. L. Baeza J. Denu J. M. (2013). Activation of the protein deacetylase SIRT6 by long-chain fatty acids and widespread deacylation by mammalian sirtuins. *J. Biol. Chem.* 288 31350–31356. 10.1074/jbc.C113.511261 24052263 PMC3829447

[B54] Ferry D. R. Smith A. Malkhandi J. Fyfe D. W. deTakats P. G. Anderson D. (1996). Phase I clinical trial of the flavonoid quercetin: pharmacokinetics and evidence for *in vivo* tyrosine kinase inhibition. *Clin. Cancer Res.* 2 659–668. 9816216

[B55] Gertz M. Fischer F. Nguyen G. T. Lakshminarasimhan M. Schutkowski M. Weyand M. (2013). Ex-527 inhibits Sirtuins by exploiting their unique NAD+-dependent deacetylation mechanism. *Proc. Natl. Acad. Sci. U.S.A.* 110 E2772-E2781. 10.1073/pnas.1303628110 23840057 PMC3725051

[B56] Gliemann L. Schmidt J. F. Olesen J. Biensø R. S. Peronard S. L. Grandjean S. U. (2013). Resveratrol blunts the positive effects of exercise training on cardiovascular health in aged men. *J. Physiol.* 591 5047–5059. 10.1113/jphysiol.2013.258061 23878368 PMC3810808

[B57] Goh K. P. Lee H. Y. Lau D. P. Supaat W. Chan Y. H. Koh A. F. (2014). Effects of resveratrol in patients with type 2 diabetes mellitus on skeletal muscle SIRT1 expression and energy expenditure. *Int. J. Sport Nutr. Exerc. Metab.* 24 2–13. 10.1123/ijsnem.2013-0045 23918588

[B58] Grozinger C. M. Chao E. D. Blackwell H. E. Moazed D. Schreiber S. L. (2001). Identification of a class of small molecule inhibitors of the sirtuin family of NAD-dependent deacetylases by phenotypic screening. *J. Biol. Chem.* 276 38837–38843. 10.1074/jbc.M106779200 11483616

[B59] Guarente L. Picard F. (2005). Calorie restriction–the SIR2 connection. *Cell* 120 473–482. 10.1016/j.cell.2005.01.029 15734680

[B60] Gugler R. Leschik M. Dengler H. J. (1975). Disposition of quercetin in man after single oral and intravenous doses. *Eur. J. Clin. Pharmacol.* 9 (2-3), 229–234. 10.1007/BF00614022 1233267

[B61] Harper S. A. Bassler J. R. Peramsetty S. Yang Y. Roberts L. M. Drummer D. (2021). Resveratrol and exercise combined to treat functional limitations in late life: a pilot randomized controlled trial. *Exp. Gerontol.* 143:111111. 33068691 10.1016/j.exger.2020.111111 PMC7855904

[B62] Heebøll S. Kreuzfeldt M. Hamilton-Dutoit S. Kjaer Poulsen M. Stødkilde-Jørgensen H. Møller H. J. (2016). Placebo-controlled, randomised clinical trial: high-dose resveratrol treatment for non-alcoholic fatty liver disease. *Scand. J. Gastroenterol.* 51 456–464. 26784973 10.3109/00365521.2015.1107620

[B63] Herranz D. Maraver A. Cañamero M. Gómez-López G. Inglada-Pérez L. Robledo M. (2013). SIRT1 promotes thyroid carcinogenesis driven by PTEN deficiency. *Oncogene* 32 4052–4056. 10.1038/onc.2012.407 22986535

[B64] Herskovits A. Z. Guarente L. (2014). SIRT1 in neurodevelopment and brain senescence. *Neuron* 81 471–483. 10.1016/j.neuron.2014.01.028 24507186 PMC4040287

[B65] Hodgin K. S. Donovan E. K. Kekes-Szabo S. Lin J. C. Feick J. Massey R. L. (2021). A placebo-controlled, pseudo-randomized, crossover trial of botanical agents for gulf war illness: resveratrol (*Polygonum cuspidatum*), Luteolin, and Fisetin (*Rhus succedanea*). *Int. J. Environ. Res. Public Health* 18:2483. 10.3390/ijerph18052483 33802381 PMC7967624

[B66] Hoffmann E. Wald J. Lavu S. Roberts J. Beaumont C. Haddad J. (2013). Pharmacokinetics and tolerability of SRT2104, a first-in-class small molecule activator of SIRT1, after single and repeated oral administration in man. *Br. J. Clin. Pharmacol.* 75 186–196. 10.1111/j.1365-2125.2012.04340.x 22616762 PMC3555058

[B67] Hoseini A. Namazi G. Farrokhian A. Reiner Ž. Aghadavod E. Bahmani F. (2019). The effects of resveratrol on metabolic status in patients with type 2 diabetes mellitus and coronary heart disease. *Food Funct*. 10 6042–6051. 10.1039/c9fo01075k 31486447

[B68] Hou X. Rooklin D. Fang H. Zhang Y. (2016). Resveratrol serves as a protein-substrate interaction stabilizer in human SIRT1 activation. *Sci. Rep.* 6:38186. 10.1038/srep38186 27901083 PMC5128864

[B69] Howells L. M. Berry D. P. Elliott P. J. Jacobson E. W. Hoffmann E. Hegarty B. (2011). Phase I randomized, double-blind pilot study of micronized resveratrol (SRT501) in patients with hepatic metastases–safety, pharmacokinetics, and pharmacodynamics. *Cancer Prev. Res.* 4 1419–1425. 10.1158/1940-6207.CAPR-11-0148 21680702 PMC3173869

[B70] Howitz K. T. Bitterman K. J. Cohen H. Y. Lamming D. W. Lavu S. Wood J. G. (2003). Small molecule activators of sirtuins extend *Saccharomyces cerevisiae* lifespan. *Nature* 425 191–196. 10.1038/nature01960 12939617

[B71] Hu T. Lu X. Y. Shi J. J. Liu X. Q. Chen Q. B. Wang Q. (2020). Quercetin protects against diabetic encephalopathy via SIRT1/NLRP3 pathway in db/db mice. *J. Cell. Mol. Med.* 24 3449–3459. 10.1111/jcmm.15026 32000299 PMC7131910

[B72] Hubbard B. P. Gomes A. P. Dai H. Li J. Case A. W. Considine T. (2013). Evidence for a common mechanism of SIRT1 regulation by allosteric activators. *Science* 339 1216–1219. 10.1126/science.1231097 23471411 PMC3799917

[B73] Huhn S. Beyer F. Zhang R. Lampe L. Grothe J. Kratzsch J. (2018). Effects of resveratrol on memory performance, hippocampus connectivity and microstructure in older adults – A randomized controlled trial. *Neuroimage* 174 177–190. 29548848 10.1016/j.neuroimage.2018.03.023

[B74] Iannitti R. G. Floridi A. Lazzarini A. Tantucci A. Russo R. Ragonese F. (2020). Resveratrol supported on magnesium DiHydroxide (Resv@MDH) represents an oral formulation of resveratrol with better gastric absorption and bioavailability respect to pure resveratrol. *Front. Nutr.* 7:570047. 10.3389/fnut.2020.570047 34422874 PMC8377765

[B75] Imai S. Armstrong C. M. Kaeberlein M. Guarente L. (2000). Transcriptional silencing and longevity protein Sir2 is an NAD-dependent histone deacetylase. *Nature* 403 795–800. 10693811 10.1038/35001622

[B76] Ittner L. M. Götz J. (2011). Amyloid-β and tau — a toxic pas de deux in Alzheimer’s disease. *Nat. Rev. Neurosci.* 12 67–72. 10.1038/nrn2967 21193853

[B77] Jaiswal A. Xudong Z. Zhenyu J. Saretzki G. (2021). Mitochondrial sirtuins in stem cells and cancer. *FEBS J*. 10.1111/febs.15879 [Epub ahead of print]. 33866670

[B78] Jang M. Cai L. Udeani G. O. Slowing K. V. Thomas C. F. Beecher C. W. W. (1997). Cancer chemopreventive activity of resveratrol, a natural product derived from grapes. *Science* 275 218–220. 8985016 10.1126/science.275.5297.218

[B79] Jeong H. Then F. Melia T. J. Jr Mazzulli J. R. Cui L. Savas J. N. (2009). Acetylation targets mutant huntingtin to autophagosomes for degradation. *Cell* 137 60–72. 10.1016/j.cell.2009.03.018 19345187 PMC2940108

[B80] Jiang H. Khan S. Wang Y. Charron G. He B. Sebastian C. (2013). SIRT6 regulates TNF-alpha secretion through hydrolysis of long-chain fatty acyl lysine. *Nature* 496 110–113. 10.1038/nature12038 23552949 PMC3635073

[B81] Johnson J. J. Nihal M. Siddiqui I. A. Scarlett C. O. Bailey H. H. Mukhtar H. (2011). Enhancing the bioavailability of resveratrol by combining it with piperine. *Mol. Nutr. Food Res.* 55 1169–1176. 21714124 10.1002/mnfr.201100117 PMC3295233

[B82] Kaeberlein M. McDonagh T. Heltweg B. Hixon J. Westman E. A. Caldwell S. D. (2005). Substrate-specific activation of sirtuins by resveratrol. *J. Biol. Chem*. 280 17038–17045. 15684413 10.1074/jbc.M500655200

[B83] Kantartzis K. Fritsche L. Bombrich M. Machann J. Schick F. Staiger H. (2018). Effects of resveratrol supplementation on liver fat content in overweight and insulin-resistant subjects: a randomized, double-blind, placebo-controlled clinical trial. *Diabetes Obes. Metab*. 20 1793–1797. 10.1111/dom.13268 29484808

[B84] KaŞıkcı M. B. Bağdatlıoğlu N. (2016). Bioavailability of quercetin. *Curr. Res. Nutr. Food Sci. J.* 4 146–151.

[B85] Kennedy D. O. Wightman E. L. Reay J. L. Lietz G. Okello E. J. Wilde A. (2010). Effects of resveratrol on cerebral blood flow variables and cognitive performance in humans: a double-blind, placebo-controlled, crossover investigation. *Am. J. Clin. Nutr.* 91 1590–1597. 10.3945/ajcn.2009.28641 20357044

[B86] Kjaer T. N. Ornstrup M. J. Poulsen M. M. Stødkilde-Jørgensen H. Jessen N. Jørgensen J. O. L. (2017). No beneficial effects of resveratrol on the metabolic syndrome: a randomized placebo-controlled clinical trial. *J. Clin. Endocrinol. Metab.* 102 1642–1651. 28182820 10.1210/jc.2016-2160

[B87] Knop F. K. Konings E. Timmers S. Schrauwen P. Holst J. J. Blaak E. E. (2013). Thirty days of resveratrol supplementation does not affect postprandial incretin hormone responses, but suppresses postprandial glucagon in obese subjects. *Diabet. Med.* 30 1214–1218. 23663119 10.1111/dme.12231

[B88] Köbe T. Witte A. V. Schnelle A. Tesky V. A. Pantel J. Schuchardt J.-P. (2017). Impact of resveratrol on glucose control, hippocampal structure and connectivity, and memory performance in patients with mild cognitive impairment. *Front. Neurosci.* 11:105. 10.3389/fnins.2017.00105 28326010 PMC5339301

[B89] Kosciuk T. Wang M. Hong J. Y. Lin H. (2019). Updates on the epigenetic roles of sirtuins. *Curr. Opin. Chem. Biol*. 51 18–29. 30875552 10.1016/j.cbpa.2019.01.023 PMC6698398

[B90] Krueger J. G. Suárez-Fariñas M. Cueto I. Khacherian A. Matheson R. Parish L. C. (2015). A randomized, placebo-controlled study of SRT2104, a SIRT1 activator, in patients with moderate to severe psoriasis. *PLoS One* 10:e0142081. 10.1371/journal.pone.0142081 26556603 PMC4640558

[B91] Kumar S. Lombard D. B. (2018). Functions of the sirtuin deacylase SIRT5 in normal physiology and pathobiology. *Crit. Rev. Biochem. Mol. Biol.* 53 311–334. 10.1080/10409238.2018.1458071 29637793 PMC6233320

[B92] la Porte C. Voduc N. Zhang G. Seguin I. Tardiff D. Singhal N. (2010). Steady-State pharmacokinetics and tolerability of trans-resveratrol 2000 mg twice daily with food, quercetin and alcohol (ethanol) in healthy human subjects. *Clin. Pharmacokinet.* 49 449–454. 10.2165/11531820-000000000-00000 20528005

[B93] Lakshminarasimhan M. Rauh D. Schutkowski M. Steegborn C. (2013). SIRT1 activation by resveratrol is substrate sequence-selective. *Aging* 5 151–154. 10.18632/aging.100542 23524286 PMC3629287

[B94] Landry J. Sutton A. Tafrov S. T. Heller R. C. Stebbins J. Pillus L. (2000). The silencing protein SIR2 and its homologs are NAD-dependent protein deacetylases. *Proc. Natl. Acad. Sci. U.S.A*. 97 5807–5811. 10811920 10.1073/pnas.110148297 PMC18515

[B95] Lesjak M. Beara I. Simin N. Pintać D. Majkić T. Bekvalac K. (2018). Antioxidant and anti-inflammatory activities of quercetin and its derivatives. *J. Funct. Foods* 40 68–75.

[B96] Leyton L. Hott M. Acuña F. Caroca J. Nuñez M. Martin C. (2015). Nutraceutical activators of AMPK/Sirt1 axis inhibit viral production and protect neurons from neurodegenerative events triggered during HSV-1 infection. *Virus Res.* 205 63–72. 10.1016/j.virusres.2015.05.015 26031763

[B97] Libri V. Brown A. P. Gambarota G. Haddad J. Shields G. S. Dawes H. (2012). A pilot randomized, placebo controlled, double blind phase i trial of the novel SIRT1 activator SRT2104 in elderly volunteers. *PLoS One* 7:e51395. 10.1371/journal.pone.0051395 23284689 PMC3527451

[B98] Lipinski C. A. Lombardo F. Dominy B. W. Feeney P. J. (2001). Experimental and computational approaches to estimate solubility and permeability in drug discovery and development settings. *Adv. Drug Deliv. Rev.* 46 (1-3), 3–26. 10.1016/s0169-409x(00)00129-0 11259830

[B99] Liu T. Yang Q. Zhang X. Qin R. Shan W. Zhang H. (2020). Quercetin alleviates kidney fibrosis by reducing renal tubular epithelial cell senescence through the SIRT1/PINK1/mitophagy axis. *Life Sci.* 257:118116. 10.1016/j.lfs.2020.118116 32702447

[B100] Løkken N. Khawajazada T. Storgaard J. Raaschou-Pedersen D. Ørngreen M. Vissing J. (2019). P.58No effect of resveratrol supplementation in patients with mitochondrial myopathy - a randomized, double-blind, placebo-controlled, cross-over study. *Neuromuscul. Disord.* 29 S57–S58.

[B101] Lugrin J. Ciarlo E. Santos A. Grandmaison G. dos Santos I. Le Roy D. (2013). The sirtuin inhibitor cambinol impairs MAPK signaling, inhibits inflammatory and innate immune responses and protects from septic shock. *Biochim. Biophys. Acta* 1833 1498–1510. 10.1016/j.bbamcr.2013.03.004 23499872

[B102] Luo J. Nikolaev A. Y. Imai S.-I. Chen D. Su F. Shiloh A. (2001). Negative Control of p53 by Sir2α promotes cell survival under stress. *Cell* 107 137–148. 11672522 10.1016/s0092-8674(01)00524-4

[B103] Lutz M. I. Milenkovic I. Regelsberger G. Kovacs G. G. (2014). Distinct patterns of sirtuin expression during progression of Alzheimer’s disease. *Neuromol. Med*. 16 405–414. 10.1007/s12017-014-8288-8 24464653

[B104] Magar R. T. Sohng J. K. (2020). A review on structure, modifications and structure-activity relation of quercetin and its derivatives. *J. Microbiol. Biotechnol.* 30 11–20. 10.4014/jmb.1907.07003 31752056 PMC9728256

[B105] Mai A. Massa S. Lavu S. Pezzi R. Simeoni S. Ragno R. (2005). Design, synthesis, and biological evaluation of sirtinol analogues as class III histone/protein deacetylase (Sirtuin) inhibitors. *J. Med. Chem.* 48 7789–7795. 10.1021/jm050100l 16302818

[B106] Mankowski R. T. You L. Buford T. W. Leeuwenburgh C. Manini T. M. Schneider S. (2020). Higher dose of resveratrol elevated cardiovascular disease risk biomarker levels in overweight older adults – A pilot study. *Exp. Gerontol.* 131:110821. 10.1016/j.exger.2019.110821 31891746 PMC8168448

[B107] Manna S. K. Mukhopadhyay A. Aggarwal B. B. (2000). Resveratrol suppresses TNF-induced activation of nuclear transcription factors NF-kappa B, activator protein-1, and apoptosis: potential role of reactive oxygen intermediates and lipid peroxidation. *J. Immunol.* 164 6509–6519. 10.4049/jimmunol.164.12.6509 10843709

[B108] Marques B. Trindade M. Aquino J. C. F. Cunha A. R. Gismondi R. O. Neves M. F. (2018). Beneficial effects of acute trans-resveratrol supplementation in treated hypertensive patients with endothelial dysfunction. *Clin. Exp. Hypertens.* 40 218–223. 10.1080/10641963.2017.1288741 29431520

[B109] Massi A. Bortolini O. Ragno D. Bernardi T. Sacchetti G. Tacchini M. (2017). Research progress in the modification of quercetin leading to anticancer agents. *Molecules* 22:1270. 10.3390/molecules22081270 PMC6152094 28758919

[B110] McCallum S. Wald J. Hoffmann E. Jayaramachandran S. Bhatt K. Englehart S. (2014). “Modified Release Formulations Do Not Enhance the PK of the Novel SIRT1 Activator SRT2104 (GSK2245840B),” in *Proceedings of the 2014 Annual Meeting & Exposition - American Association of Pharmaceutical Scientists*, San Diego, CA.

[B111] McDermott M. M. Leeuwenburgh C. Guralnik J. M. Tian L. Sufit R. Zhao L. (2017). Effect of resveratrol on walking performance in older people with peripheral artery disease: the RESTORE randomized clinical trial. *JAMA Cardiol.* 2 902–907. 10.1001/jamacardio.2017.0538 28403379 PMC5815080

[B112] Mehboob H. Tahir I. M. Iqbal T. Saleem S. Perveen S. Farooqi A. (2017). Effect of UDP-Glucuronosyltransferase (UGT) 1A Polymorphism (rs8330 and rs10929303) on Glucuronidation Status of Acetaminophen. *Dose Response* 15:1559325817723731. 10.1177/1559325817723731 28932176 PMC5598801

[B113] Mendes da Silva D. Gross L. A. Neto E. D. P. G. Lessey B. A. Savaris R. F. (2017). The use of resveratrol as an adjuvant treatment of pain in endometriosis: a randomized clinical trial. *J. Endocr. Soc.* 1 359–369. 10.1210/js.2017-00053 29264492 PMC5686687

[B114] Méndez-del Villar M. González-Ortiz M. Martínez-Abundis E. Pérez-Rubio K. G. Lizárraga-Valdez R. (2014). Effect of resveratrol administration on metabolic syndrome, insulin sensitivity, and insulin secretion. *Metab. Syndr. Relat. Disord.* 12 497–501. 25137036 10.1089/met.2014.0082

[B115] Miller J. J. Fink A. Banagis J. A. Nagashima H. Subramanian M. Lee C. K. (2021). Sirtuin activation targets IDH-mutant tumors. *Neuro Oncol.* 23 53–62. 10.1093/neuonc/noaa180 32710757 PMC7850026

[B116] Milne J. C. Lambert P. D. Schenk S. Carney D. P. Smith J. J. Gagne D. J. (2007). Small molecule activators of SIRT1 as therapeutics for the treatment of type 2 diabetes. *Nature* 450 712–716. 18046409 10.1038/nature06261 PMC2753457

[B117] Min S.-W. Cho S.-H. Zhou Y. Schroeder S. Haroutunian V. Seeley W. W. (2010). Acetylation of tau inhibits its degradation and contributes to tauopathy. *Neuron* 67 953–966. 20869593 10.1016/j.neuron.2010.08.044 PMC3035103

[B118] Miraglia Del Giudice M. Maiello N. Decimo F. Capasso M. Campana G. Leonardi S. (2014). Resveratrol plus carboxymethyl-β-glucan may affect respiratory infections in children with allergic rhinitis. *Pediatr. Allergy Immunol.* 25 724–728. 10.1111/pai.12279 25199647

[B119] Monod J. Wyman J. Changeux J.-P. (1965). On the nature of allosteric transitions: a plausible model. *J. Mol. Biol.* 12 88–118. 14343300 10.1016/s0022-2836(65)80285-6

[B120] Moodi Z. Bagherzade G. Peters J. (2021). Quercetin as a precursor for the synthesis of novel Nanoscale Cu (II) complex as a catalyst for alcohol oxidation with high antibacterial activity. *Bioinorg. Chem. Appl.* 2021:8818452. 10.1155/2021/8818452 33747070 PMC7952193

[B121] Morselli E. Maiuri M. C. Markaki M. Megalou E. Pasparaki A. Palikaras K. (2010). Caloric restriction and resveratrol promote longevity through the Sirtuin-1-dependent induction of autophagy. *Cell Death Dis*. 1:e10. 10.1038/cddis.2009.8 21364612 PMC3032517

[B122] Moskaug J. Carlsen H. Myhrstad M. Blomhoff R. (2004). Molecular imaging of the biological effects of quercetin and quercetin-rich foods. *Mech. Ageing Dev.* 125 315–324. 10.1016/j.mad.2004.01.007 15063108

[B123] Moussa C. Hebron M. Huang X. Ahn J. Rissman R. A. Aisen P. S. (2017). Resveratrol regulates neuro-inflammation and induces adaptive immunity in Alzheimer’s disease. *J. Neuroinflammation* 14:1. 10.1186/s12974-016-0779-0 28086917 PMC5234138

[B124] Napper A. D. Hixon J. McDonagh T. Keavey K. Pons J. F. Barker J. (2005). Discovery of Indoles as Potent and Selective Inhibitors of the Deacetylase SIRT1. *J. Med. Chem*. 48 8045–8054. 16335928 10.1021/jm050522v

[B125] Noh R. M. Venkatasubramanian S. Daga S. Langrish J. Mills N. L. Lang N. N. (2017). Cardiometabolic effects of a novel SIRT1 activator, SRT2104, in people with type 2 diabetes mellitus. *Open Heart* 4:e000647. 10.1136/openhrt-2017-000647 28912956 PMC5588958

[B126] Novelle M. G. Wahl D. Diéguez C. Bernier M. de Cabo R. (2015). Resveratrol supplementation: Where are we now and where should we go? *Ageing Res. Rev.* 21 1–15. 10.1016/j.arr.2015.01.002 25625901 PMC5835393

[B127] Oboh G. Ademosun A. O. Ogunsuyi O. B. (2016). Quercetin and its role in chronic diseases. *Adv. Exp. Med. Biol.* 929 377–387. 27771934 10.1007/978-3-319-41342-6_17

[B128] Oktyabrsky O. N. Bezmaternykh K. V. Smirnova G. V. Tyulenev A. V. (2020). Biotechnology, Effect of resveratrol and quercetin on the susceptibility of *Escherichia coli* to antibiotics. *World J. Microbiol. Biotechnol.* 36:167. 10.1007/s11274-020-02934-y 33025172

[B129] Olaharski A. J. Rine J. Marshall B. L. Babiarz J. Zhang L. Verdin E. (2005). The flavoring agent dihydrocoumarin reverses epigenetic silencing and inhibits sirtuin deacetylases. *PLoS Genet*. 1:e77. 10.1371/journal.pgen.0010077 16362078 PMC1315280

[B130] Ozemek C. Hildreth K. L. Blatchford P. J. Hurt K. J. Bok R. Seals D. R. (2020). Effects of resveratrol or estradiol on postexercise endothelial function in estrogen-deficient postmenopausal women. *J. Appl. Physiol.* 128 739–747. 32134713 10.1152/japplphysiol.00488.2019 PMC7191510

[B131] Pacholec M. Bleasdale J. E. Chrunyk B. Cunningham D. Flynn D. Garofalo R. S. (2010). SRT1720, SRT2183, SRT1460, and resveratrol are not direct activators of SIRT1. *J. Biol. Chem.* 285 8340–8351. 20061378 10.1074/jbc.M109.088682 PMC2832984

[B132] Pallos J. Bodai L. Lukacsovich T. Purcell J. M. Steffan J. S. Thompson L. M. (2008). Inhibition of specific HDACs and sirtuins suppresses pathogenesis in a *Drosophila* model of Huntington’s disease. *Hum. Mol. Genet.* 17 3767–3775. 10.1093/hmg/ddn273 18762557 PMC2581431

[B133] Park D. Jeong H. Lee M. N. Koh A. Kwon O. Yang Y. R. (2016). Resveratrol induces autophagy by directly inhibiting mTOR through ATP competition. *Sci. Rep.* 6:21772. 10.1038/srep21772 26902888 PMC4763238

[B134] Park S.-J. Ahmad F. Philp A. Baar K. Williams T. Luo H. (2012). Resveratrol ameliorates aging-related metabolic phenotypes by inhibiting cAMP phosphodiesterases. *Cell* 148 421–433. 10.1016/j.cell.2012.01.017 22304913 PMC3431801

[B135] Patel K. R. Brown V. A. Jones D. J. L. Britton R. G. Hemingway D. Miller A. S. (2010). Clinical pharmacology of resveratrol and its metabolites in colorectal cancer patients. *Cancer Res.* 70 7392–7399. 20841478 10.1158/0008-5472.CAN-10-2027 PMC2948608

[B136] Pezzuto J. M. (2011). The phenomenon of resveratrol: redefining the virtues of promiscuity. *Ann. N.Y. Acad. Sci.* 1215 123–1230. 10.1111/j.1749-6632.2010.05849.x 21261650

[B137] Pollack R. M. Barzilai N. Anghel V. Kulkarni A. S. Golden A. O’Broin P. (2017). Resveratrol improves vascular function and mitochondrial number but not glucose metabolism in older adults. *J. Gerontol*. 72 1703–1709. 10.1093/gerona/glx041 28329397 PMC5861959

[B138] Popat R. Plesner T. Davies F. Cook G. Cook M. Elliott P. (2013). A phase 2 study of SRT501 (resveratrol) with bortezomib for patients with relapsed and or refractory multiple myeloma. *Br. J. Haematol.* 160 714–717. 23205612 10.1111/bjh.12154

[B139] Porcu M. Chiarugi A. (2005). The emerging therapeutic potential of sirtuin-interacting drugs: from cell death to lifespan extension. *Trends Pharmacol. Sci*. 26 94–103. 10.1016/j.tips.2004.12.009 15681027

[B140] Poulsen M. K. Nellemann B. Bibby B. M. Stødkilde-Jørgensen H. Pedersen S. B. Grønbaek H. (2018). No effect of resveratrol on VLDL-TG kinetics and insulin sensitivity in obese men with nonalcoholic fatty liver disease. *Diabetes Obes. Metab.* 20 2504–2509. 10.1111/dom.13409 29885082

[B141] Poulsen M. M. Vestergaard P. F. Clasen B. F. Radko Y. Christensen L. P. Stødkilde-Jørgensen H. (2013). High-dose resveratrol supplementation in obese men: an investigator-initiated, randomized, placebo-controlled clinical trial of substrate metabolism, insulin sensitivity, and body composition. *Diabetes* 62 1186–1195. 10.2337/db12-0975 23193181 PMC3609591

[B142] Price N. L. Gomes A. P. Ling A. J. Duarte F. V. Martin-Montalvo A. North B. J. (2012). sirt1 is required for AMPK activation and the beneficial effects of resveratrol on mitochondrial function. *Cell Metab.* 15 675–690. 22560220 10.1016/j.cmet.2012.04.003 PMC3545644

[B143] Rahnasto-Rilla M. Tyni J. Huovinen M. Jarho E. Kulikowicz T. Ravichandran S. (2018). Natural polyphenols as sirtuin 6 modulators. *Sci. Rep.* 8:4163. 29515203 10.1038/s41598-018-22388-5 PMC5841289

[B144] Reen R. K. Jamwal D. S. Taneja S. C. Koul J. L. Dubey R. K. Wiebel F. J. (1993). Impairment of UDP-glucose dehydrogenase and glucuronidation activities in liver and small intestine of rat and guinea pig *in vitro* by piperine. *Biochem. Pharmacol.* 46 229–238. 10.1016/0006-2952(93)90408-o 8347144

[B145] Riva A. Ronchi M. Petrangolini G. Bosisio S. Allegrini P. (2019). Improved oral absorption of quercetin from quercetin phytosome^®^, a new delivery system based on food grade lecithin. *Eur. J. Drug Metab. Pharmacokinet.* 44 169–177. 10.1007/s13318-018-0517-3 30328058 PMC6418071

[B146] Roggerio A. Strunz C. M. C. Pacanaro A. P. Leal D. P. Takada J. Y. Avakian S. D. (2018). Gene expression of Sirtuin-1 and endogenous secretory receptor for advanced glycation end products in healthy and slightly overweight subjects after caloric restriction and resveratrol administration. *Nutrients* 10:937. 10.3390/nu10070937 30037068 PMC6073749

[B147] Rojas Á. Del Campo J. A. Clement S. Lemasson M. García-Valdecasas M. Gil-Gómez A. (2016). Effect of quercetin on hepatitis C virus life cycle: from viral to host targets. *Sci. Rep*. 6:31777. 10.1038/srep31777 27546480 PMC4992894

[B148] Sak K. (2014). Site-specific anticancer effects of dietary flavonoid quercetin. *Nutr. Cancer* 66 177–193. 24377461 10.1080/01635581.2014.864418

[B149] Saldanha J. F. Leal V. O. Rizzetto F. Grimmer G. H. Ribeiro-Alves M. Daleprane J. B. (2016). Effects of Resveratrol Supplementation in Nrf2 and NF-κB Expressions in nondialyzed chronic kidney disease patients: a randomized, double-blind, placebo-controlled, crossover clinical trial. *J. Ren. Nutr.* 26 401–406. 10.1053/j.jrn.2016.06.005 27523436

[B150] Sands B. E. Joshi S. Haddad J. Freudenberg J. M. Oommen D. E. Hoffmann E. (2016). Assessing colonic exposure, safety, and clinical activity of SRT2104, a Novel Oral SIRT1 activator, in patients with mild to moderate ulcerative colitis. *Inflamm. Bowel Dis.* 22 607–614. 10.1097/MIB.0000000000000597 26595549 PMC4885523

[B151] Sauve A. A. Wolberger C. Schramm V. L. Boeke J. D. (2006). The biochemistry of sirtuins. *Annu. Rev. Biochem*. 75 435–465. 16756498 10.1146/annurev.biochem.74.082803.133500

[B152] Schmidt C. (2010). GSK/Sirtris compounds dogged by assay artifacts. *Nat. Biotechnol.* 28 185–186. 10.1038/nbt0310-185 20212464

[B153] Shahbazian M. D. Grunstein M. (2007). Functions of site-specific histone acetylation and deacetylation. *Annu. Rev. Biochem*. 76 75–100. 17362198 10.1146/annurev.biochem.76.052705.162114

[B154] Sinaga S. Sudarmi S. Iksen I. Kevin K. Sari M. J. (2017). Evaluation of total phenolic, flavonoid content, antioxidant and *in vitro* antilithogenesis activities of chives leaf (*Allium schoenoprasum*, L.). *Rasayan J. Chem.* 11 1604–1608.

[B155] Singh C. K. Ahmad N. (2015). Abstract 2801: resveratrol-Quercetin combination significantly inhibits prostate cancer in TRAMP mice. *Cancer Res*. 75 (15 Supplement):2801.

[B156] Smith A. J. Oertle J. Warren D. Prato D. (2016). Quercetin: a promising flavonoid with a dynamic ability to treat various diseases, infections, and cancers. *J. Cancer Ther.* 7 83–95.

[B157] Smith M. R. Syed A. Lukacsovich T. Purcell J. Barbaro B. A. Worthge S. A. (2014). A potent and selective Sirtuin 1 inhibitor alleviates pathology in multiple animal and cell models of Huntington’s disease. *Hum. Mol. Genet.* 23 2995–3007. 24436303 10.1093/hmg/ddu010 PMC4031626

[B158] Spogli R. Bastianini M. Ragonese F. Iannitti R. Monarca L. Bastioli F. (2018). Solid dispersion of resveratrol supported on magnesium DiHydroxide (Resv@MDH) microparticles improves oral bioavailability. *Nutrients* 10:1925. 10.3390/nu10121925 PMC6315708 30563110

[B159] Springer M. Moco S. (2019). Resveratrol and its human metabolites-effects on metabolic health and obesity. *Nutrients* 11:143. 10.3390/nu11010143 PMC6357128 30641865

[B160] St Leger A. S. Cochrane A. L. Moore F. (1979). Factors associated with cardiac mortality in developed countries with particular reference to the consumption of wine. *Lancet* 1 (8124), 1017–1020. 86728 10.1016/s0140-6736(79)92765-x

[B161] Sussmuth S. D. Haider S. Landwehrmeyer G. B. Farmer R. Frost C. Tripepi G. (2015). An exploratory double-blind, randomized clinical trial with selisistat, a SirT1 inhibitor, in patients with Huntington’s disease. *Br. J. Clin. Pharmacol.* 79 465–476. 25223731 10.1111/bcp.12512 PMC4345957

[B162] Tabrizi S. J. Flower M. D. Ross C. A. Wild E. J. (2020). Huntington disease: new insights into molecular pathogenesis and therapeutic opportunities. *Nat. Rev. Neurol.* 16 529–546. 32796930 10.1038/s41582-020-0389-4

[B163] Tang J. Lu L. Liu Y. Ma J. Yang L. Li L. (2019). Quercetin improve ischemia/reperfusion-induced cardiomyocyte apoptosis *in vitro* and *in vivo* study via SIRT1/PGC-1α signaling. *J. Cell. Biochem*. 120 9747–9757. 30656723 10.1002/jcb.28255

[B164] Timmers S. de Ligt M. Phielix E. van de Weijer T. Hansen J. Moonen-Kornips E. (2016). Resveratrol as add-on therapy in subjects with well-controlled type 2 diabetes: a randomized controlled trial. *Diabetes Care* 39 2211–2217. 27852684 10.2337/dc16-0499

[B165] Trapp J. Jung M. (2006). The role of NAD+ dependent histone deacetylases (sirtuins) in ageing. *Curr. Drug Targets* 7 1553–1560. 17100594 10.2174/1389450110607011553

[B166] Turner R. S. Thomas R. G. Craft S. van Dyck C. H. Mintzer J. Reynolds B. A. (2015). A randomized, double-blind, placebo-controlled trial of resveratrol for Alzheimer disease. *Neurology* 85 1383–1391. 10.1212/WNL.0000000000002035 26362286 PMC4626244

[B167] Ung D. Nagar S. (2007). Variable sulfation of dietary polyphenols by recombinant human sulfotransferase (SULT) 1A1 genetic variants and SULT1E1. *Drug Metab. Dispos.* 35 740–746. 10.1124/dmd.106.013987 17293380

[B168] van der Made S. M. Plat J. Mensink R. P. (2015). Resveratrol does not influence metabolic risk markers related to cardiovascular health in overweight and slightly obese subjects: a randomized, placebo-controlled crossover trial. *PLoS One* 10:e0118393. 10.1371/journal.pone.0118393 25790328 PMC4366169

[B169] van der Meer A. J. Scicluna B. P. Moerland P. D. Lin J. Jacobson E. W. Vlasuk G. P. (2015). The selective Sirtuin 1 activator SRT2104 reduces endotoxin-induced cytokine release and coagulation activation in humans. *Crit. Care Med.* 43 e199–e202. 10.1097/CCM.0000000000000949 25978169

[B170] Vaz-da-Silva M. Loureiro A. I. Falcao A. Nunes T. Rocha J. F. Fernandes-Lopes C. (2008). Effect of food on the pharmacokinetic profile of trans-resveratrol. *Int. J. Clin. Pharmacol. Ther.* 46 564–570. 10.5414/CPP46564 19000554

[B171] Venkatasubramanian S. Noh R. M. Daga S. Langrish J. P. Joshi N. V. Mills N. L. (2013). Cardiovascular effects of a novel SIRT1 activator, SRT2104, in otherwise healthy cigarette smokers. *J. Am. Heart Assoc.* 2:e000042. 10.1161/JAHA.113.000042 23770971 PMC3698759

[B172] Venkatasubramanian S. Noh R. M. Daga S. Langrish J. P. Mills N. L. Waterhouse B. R. (2016). Effects of the small molecule SIRT1 activator, SRT2104 on arterial stiffness in otherwise healthy cigarette smokers and subjects with type 2 diabetes mellitus. *Open Heart* 3:e000402. 10.1136/openhrt-2016-000402 27239324 PMC4879341

[B173] Vors C. Couillard C. Paradis M. E. Gigleux I. Marin J. Vohl M. C. (2018). Supplementation with resveratrol and curcumin does not affect the inflammatory response to a high-fat meal in older adults with abdominal obesity: a randomized, placebo-controlled crossover trial. *J. Nutr.* 148 379–388. 10.1093/jn/nxx072 29546309

[B174] Walker J. M. Eckardt P. Aleman J. O. da Rosa J. C. Liang Y. Iizumi T. (2018). The effects of trans-resveratrol on insulin resistance, inflammation, and microbiota in men with the metabolic syndrome: a pilot randomized, placebo-controlled clinical trial. *J. Clin. Transl. Res.* 4 122–135. 30873501 PMC6412609

[B175] Walle T. Hsieh F. DeLegge M. H. Oatis J. E. Jr Walle U. K. (2004). High absorption but very low bioavailability of oral resveratrol in humans. *Drug Metab. Dispos.* 32 1377–1382. 10.1124/dmd.104.000885 15333514

[B176] Wang S. Yao J. Zhou B. Yang J. Chaudry M. T. Wang M. (2018). Bacteriostatic effect of quercetin as an antibiotic alternative *in vivo* and its antibacterial mechanism *in vitro*. *J. Food Prot.* 81 68–78. 10.4315/0362-028X.JFP-17-214 29271686

[B177] Wang Z. Yuan H. Roth M. Stark J. M. Bhatia R. Chen W. Y. (2013). SIRT1 deacetylase promotes acquisition of genetic mutations for drug resistance in CML cells. *Oncogene* 32 589–598. 10.1038/onc.2012.83 22410779 PMC3376246

[B178] Watroba M. Dudek I. Skoda M. Stangret A. Rzodkiewicz P. Szukiewicz D. (2017). Sirtuins, epigenetics and longevity. *Ageing Res. Rev.* 40 11–19. 10.1016/j.arr.2017.08.001 28789901

[B179] Westerberg G. Chiesa J. A. Andersen C. A. Diamanti D. Magnoni L. Pollio G. (2015). Safety, pharmacokinetics, pharmacogenomics and QT concentration-effect modelling of the SirT1 inhibitor selisistat in healthy volunteers. *Br. J. Clin. Pharmacol.* 79 477–491. 10.1111/bcp.12513 25223836 PMC4345958

[B180] Wiewel M. A. van der Meer A. J. Haddad J. Jacobson E. W. Vlasuk G. P. van der Poll T. (2013). SRT2379, a small-molecule SIRT1 activator, fails to reduce cytokine release in a human endotoxemia model. *Crit. Care* 17:8. 10.1186/cc12909

[B181] Wightman E. L. Reay J. L. Haskell C. F. Williamson G. Dew T. P. Kennedy D. O. (2014). Effects of resveratrol alone or in combination with piperine on cerebral blood flow parameters and cognitive performance in human subjects: a randomised, double-blind, placebo-controlled, cross-over investigation. *Br. J. Nutr.* 112 203–213. 10.1017/S0007114514000737 24804871

[B182] Wightman E. Eschle T. M. Kennedy D. (2019). The Cognitive effects of the polyphenol resveratrol in young, healthy humans: a review of six balanced crossover, placebo controlled, double blind trials. *Int. J. Nutr. Health Food Saf.* 1 001–009.

[B183] Wong G. He S. Siragam V. Bi Y. Mbikay M. Chretien M. (2017). Antiviral activity of quercetin-3-β-OD-glucoside against Zika virus infection. *Virol. Sin.* 32 545–547. 10.1007/s12250-017-4057-9 28884445 PMC6598929

[B184] Wu Y. Li X. Zhu J. X. Xie W. Le W. Fan Z. (2011). Resveratrol-activated AMPK/SIRT1/autophagy in cellular models of Parkinson’s disease. *Neurosignals* 19 163–174. 10.1159/000328516 21778691 PMC3699815

[B185] Yiu E. Tai G. Peverill R. Lee K. Croft K. Mori T. (2013). An open label clinical pilot study of resveratrol as a treatment for friedreich ataxia (S43.006). *Neurology* 80 (7 Suppl.):S43.006.

[B186] Yoo J. Y. Kim T. H. Fazleabas A. T. Palomino W. A. Ahn S. H. Tayade C. (2017). KRAS Activation and over-expression of SIRT1/BCL6 Contributes to the Pathogenesis of Endometriosis and Progesterone Resistance. *Sci. Rep.* 7:6765. 28754906 10.1038/s41598-017-04577-w PMC5533722

[B187] Yoshino J. Conte C. Fontana L. Mittendorfer B. Imai S.-I. Schechtman K. B. (2012). Resveratrol supplementation does not improve metabolic function in nonobese women with normal glucose tolerance. *Cell Metab.* 16 658–664. 10.1016/j.cmet.2012.09.015 23102619 PMC3496026

[B188] You W. Zheng W. Weiss S. Chua K. F. Steegborn C. (2019). Structural basis for the activation and inhibition of Sirtuin 6 by quercetin and its derivatives. *Sci. Rep.* 9:19176. 10.1038/s41598-019-55654-1 31844103 PMC6914789

[B189] Yuan H. Marmorstein R. (2012). Structural basis for sirtuin activity and inhibition. *J. Biol. Chem.* 287 42428–42435. 10.1074/jbc.R112.372300 23086949 PMC3522243

[B190] Zeng Y. Nikitkova A. Abdelsalam H. Li J. Xiao J. (2019). Activity of quercetin and kaemferol against Streptococcus mutans biofilm. *Arch Oral Biol.* 98 9–16. 10.1016/j.archoralbio.2018.11.005 30419487 PMC6555400

[B191] Zhang J. Y. Holbrook M. Inagaki E. Feng B. Ko D. Weisbrod R. (2017). The effects of resveratrol treatment on vascular function in type 2 diabetes mellitus. *Arterioscler. Thromb. Vasc. Biol.* 37:A164. 10.1161/atvb.37.suppl_1.164

[B192] Zhu C. W. Grossman H. Neugroschl J. Parker S. Burden A. Luo X. Sano M. (2018). A randomized, double-blind, placebo-controlled trial of resveratrol with glucose and malate (RGM) to slow the progression of Alzheimer’s disease: a pilot study. *Alzheimers Dement.* 4 609–616. 10.1016/j.trci.2018.09.009 30480082 PMC6240843

[B193] Zocchi L. Sassone-Corsi P. (2012). SIRT1-mediated deacetylation of MeCP2 contributes to BDNF expression. *Epigenetics* 7 695–700. 10.4161/epi.20733 22677942 PMC3414390

[B194] Zondervan K. T. Becker C. M. Missmer S. A. (2020). Endometriosis. *N. Engl. J. Med.* 382 1244–1256. 10.1056/NEJMra1810764 32212520

[B195] Zortea K. Franco V. C. Guimarães P. Belmonte-de-Abreu P. S. (2016). Resveratrol supplementation did not improve cognition in patients with schizophrenia: results from a randomized clinical trial. *Front. Psychiatry* 7:159. 10.3389/fpsyt.2016.00159 27695423 PMC5025441

